# Identification of genetic elements in metabolism by high-throughput mouse phenotyping

**DOI:** 10.1038/s41467-017-01995-2

**Published:** 2018-01-18

**Authors:** Jan Rozman, Birgit Rathkolb, Manuela A. Oestereicher, Christine Schütt, Aakash Chavan Ravindranath, Stefanie Leuchtenberger, Sapna Sharma, Martin Kistler, Monja Willershäuser, Robert Brommage, Terrence F. Meehan, Jeremy Mason, Hamed Haselimashhadi, IMPC Consortium, Antonio Aguilar-Pimentel, Lore Becker, Irina Treise, Kristin Moreth, Lillian Garrett, Sabine M. Hölter, Annemarie Zimprich, Susan Marschall, Oana V. Amarie, Julia Calzada-Wack, Frauke Neff, Laura Brachthäuser, Christoph Lengger, Claudia Stoeger, Lilly Zapf, Yi-Li Cho, Patricia da Silva-Buttkus, Markus J. Kraiger, Philipp Mayer-Kuckuk, Karen Kristine Gampe, Moya Wu, Nathalie Conte, Jonathan Warren, Chao-Kung Chen, Ilinca Tudose, Mike Relac, Peter Matthews, Heather L. Cater, Helen P. Natukunda, James Cleak, Lydia M. Teboul, Sharon Clementson-Mobbs, Zsombor Szoke-Kovacs, Alison P. Walling, Sara J. Johnson, Gemma F. Codner, Tanja Fiegel, Natalie Ring, Henrik Westerberg, Simon Greenaway, Duncan Sneddon, Hugh Morgan, Jorik Loeffler, Michelle E. Stewart, Ramiro Ramirez-Solis, Allan Bradley, William C. Skarnes, Karen P. Steel, Simon A. Maguire, Joshua Dench, David Lafont, Valerie E. Vancollie, Selina A. Pearson, Amy S. Gates, Mark Sanderson, Carl Shannon, Lauren F. E. Anthony, Maksymilian T. Sumowski, Robbie S. B. McLaren, Brendan Doe, Hannah Wardle-Jones, Mark N. D. Griffiths, Antonella Galli, Agnieszka Swiatkowska, Christopher M. Isherwood, Anneliese O. Speak, Emma L. Cambridge, Heather M. Wilson, Susana S. Caetano, Anna Karin B. Maguire, David J. Adams, Joanna Bottomley, Ed Ryder, Diane Gleeson, Laurent Pouilly, Stephane Rousseau, Aurélie Auburtin, Patrick Reilly, Abdel Ayadi, Mohammed Selloum, Joshua A. Wood, Dave Clary, Peter Havel, Todd Tolentino, Heather Tolentino, Mike Schuchbauer, Sheryl Pedroia, Amanda Trainor, Esi Djan, Milton Pham, Alison Huynh, Vincent De Vera, John Seavitt, Juan Gallegos, Arturo Garza, Elise Mangin, Joel Senderstrom, Iride Lazo, Kate Mowrey, Ritu Bohat, Rodney Samaco, Surabi Veeraragavan, Christine Beeton, Sowmya Kalaga, Lois Kelsey, Igor Vukobradovic, Zorana Berberovic, Celeste Owen, Dawei Qu, Ruolin Guo, Susan Newbigging, Lily Morikawa, Napoleon Law, Xueyuan Shang, Patricia Feugas, Yanchun Wang, Mohammad Eskandarian, Yingchun Zhu, Patricia Penton, Valerie Laurin, Shannon Clarke, Qing Lan, Gillian Sleep, Amie Creighton, Elsa Jacob, Ozge Danisment, Marina Gertsenstein, Monica Pereira, Suzanne MacMaster, Sandra Tondat, Tracy Carroll, Jorge Cabezas, Jane Hunter, Greg Clark, Mohammed Bubshait, David Miller, Khondoker Sohel, Hibret Adissu, Milan Ganguly, Alexandr Bezginov, Francesco Chiani, Chiara Di Pietro, Gianfranco Di Segni, Olga Ermakova, Filomena Ferrara, Paolo Fruscoloni, Aalessia Gambadoro, Serena Gastaldi, Elisabetta Golini, Gina La Sala, Silvia Mandillo, Daniela Marazziti, Marzia Massimi, Rafaele Matteoni, Tiziana Orsini, Miriam Pasquini, Marcello Raspa, Aline Rauch, Gianfranco Rossi, Nicoletta Rossi, Sabrina Putti, Ferdinando Scavizzi, Giuseppe D. Tocchini-Valentini, Shigeharu Wakana, Tomohiro Suzuki, Masaru Tamura, Hideki Kaneda, Tamio Furuse, Kimio Kobayashi, Ikuo Miura, Ikuko Yamada, Yuichi Obata, Atsushi Yoshiki, Shinya Ayabe, J. Nicole Chambers, Karel Chalupsky, Claudia Seisenberger, Antje Bürger, Joachim Beig, Ralf Kühn, Andreas Hörlein, Joel Schick, Oskar Oritz, Florian Giesert, Jochen Graw, Markus Ollert, Carsten Schmidt-Weber, Tobias Stoeger, Ali Önder Yildirim, Oliver Eickelberg, Thomas Klopstock, Dirk H. Busch, Raffi Bekeredjian, Andreas Zimmer, Jules O. Jacobsen, Damian Smedley, Mary E. Dickinson, Frank Benso, Iva Morse, Hyoung-Chin Kim, Ho Lee, Soo Young Cho, Tertius Hough, Ann-Marie Mallon, Sara Wells, Luis Santos, Christopher J. Lelliott, Jacqueline K. White, Tania Sorg, Marie-France Champy, Lynette R. Bower, Corey L. Reynolds, Ann M. Flenniken, Stephen A. Murray, Lauryl M. J. Nutter, Karen L. Svenson, David West, Glauco P. Tocchini-Valentini, Arthur L. Beaudet, Fatima Bosch, Robert B. Braun, Michael S. Dobbie, Xiang Gao, Yann Herault, Ala Moshiri, Bret A. Moore, K. C. Kent Lloyd, Colin McKerlie, Hiroshi Masuya, Nobuhiko Tanaka, Paul Flicek, Helen E. Parkinson, Radislav Sedlacek, Je Kyung Seong, Chi-Kuang Leo Wang, Mark Moore, Steve D. Brown, Matthias H. Tschöp, Wolfgang Wurst, Martin Klingenspor, Eckhard Wolf, Johannes Beckers, Fausto Machicao, Andreas Peter, Harald Staiger, Hans-Ulrich Häring, Harald Grallert, Monica Campillos, Holger Maier, Helmut Fuchs, Valerie Gailus-Durner, Thomas Werner, Martin Hrabe de Angelis

**Affiliations:** 1 German Mouse Clinic, Institute of Experimental Genetics, Helmholtz Zentrum München, German Research Center for Environmental Health, Ingolstädter Landstr. 1, 85764 Neuherberg, Germany; 2 German Center for Diabetes Research (DZD), Ingolstädter Landstr. 1, 85764 Neuherberg, Germany; 3 Ludwig-Maximilians-Universität München, Gene Center, Institute of Molecular Animal Breeding and Biotechnology, Feodor-Lynen Strasse 25, 81377 Munich, Germany; 4 Institute of Bioinformatics and Systems Biology, Helmholtz Zentrum München, German Research Center for Environmental Health, Ingolstädter Landstr. 1, 85764 Neuherberg, Germany; 5 Research Unit of Molecular Epidemiology, Institute of Epidemiology II, Helmholtz Zentrum München, Ingolstädter Landstr. 1, 85764 Neuherberg, Germany; 6 Chair of Molecular Nutritional Medicine, Technical University of Munich, TUM School of Life Sciences Weihenstephan, 85354 Freising, Germany; 7 EKFZ – Else Kröner-Fresenius Center for Nutritional Medicine, Technical University of Munich, 85354 Freising, Germany; 8 ZIEL – Institute for Food & Health, Technical University of Munich, 85354 Freising, Germany; 9 European Molecular Biology Laboratory, European Bioinformatics Institute, Wellcome Genome Campus, Hinxton, Cambridge, CB10 1SD UK; 10 Medical Research Council Harwell (Mammalian Genetics Unit and Mary Lyon Centre), Oxfordshire, OX11 0RD UK; 11 The Wellcome Trust Sanger Institute, Wellcome Genome Campus, Hinxton, Cambridge, CB10 1SA UK; 12 The Jackson Laboratory, 600 Main Street, Bar Harbor, ME 04609 USA; 13 CELPHEDIA, PHENOMIN, Institut Clinique de la Souris (ICS), 1 Rue Laurent Fries, 67404 Illkirch-Graffenstaden, France; 14 Institut de Génétique et de Biologie Moléculaire et Cellulaire (IGBMC), Parc d’innovation, 1 Rue Laurent Fries - BP 10142, 67404 Illkirch, France; 15 Centre National de la Recherche Scientifique, UMR7104, 67404 Illkirch, France; 16 Institut National de la Santé et de la Recherche Médicale, U964, 67404 Illkirch, France; 17 Université de Strasbourg, 67404 Illkirch, France; 18 Mouse Biology Program, University of California, One Shields Avenue, Davis, CA 95616 USA; 19 Department of Molecular and Human Genetics, Baylor College of Medicine, 7702 Main St, Houston Medical Center, Houston, TX 77030-4406 USA; 20 The Centre for Phenogenomics, 25 Orde St, Toronto, M5T 3H7, ON Canada; 21 The Hospital for Sick Children, 600 University Avenue, Toronto, ON M5G 1X5 Canada; 22 Lunenfeld-Tanenbaum Research Institute, Mount Sinai Hospital, Joseph and Wolf Lebovic Health Complex, 600 University Avenue, Toronto, ON M5G 1X5 Canada; 23 Children’s Hospital Oakland Research Institute, 5700 Martin Luther King Jr. Way, Oakland, CA 94609 USA; 24 Monterotondo Mouse Clinic, Italian National Research Council (CNR), Institute of Cell Biology and Neurobiology, Adriano Buzzati-Traverso Campus, Via E. Ramarini 32, Monterotondo Scalo, RM 00015 Italy; 25 Center of Animal Biotechnology and Gene Therapy and Department of Biochemistry and Molecular Biology, Universitat Autònoma de Barcelona, Bellaterra, Spain; 26 Australian Phenomics Network, John Curtin School of Medical Research, Australian National University, 131 Garran Road, Canberra, ACT 2601 Australia; 27 SKL of Pharmaceutical Biotechnology and Model Animal Research Center, Collaborative Innovation Center for Genetics and Development, Nanjing Biomedical Research Institute, Nanjing University, Nanjing, 210061 China; 28 Department of Ophthalmology & Vision Science, School of Medicine, U.C. Davis, 77 Cadillac Drive, Sacramento, 95825 CA USA; 29 William R. Pritchard Veterinary Medical Teaching Hospital, School of Veterinary Medicine, U.C. Davis, One Shields Avenue, Davis, 95616 CA USA; 30 RIKEN BioResource Center, 3-1-1 Koyadai, Tsukuba Ibaraki, 305-0074 Japan; 31 Czech Centre for Phenogenomics, Institute of Molecular Genetics, Prumyslova 595, 252 50 Vestec, Czech Republic; 32 Korea Mouse Phenotyping Consortium (KMPC) and BK21 Program for Veterinary Science, Research Institute for Veterinary Science, College of Veterinary Medicine, Seoul National University, 599 Gwanangno, Gwanak-gu, Seoul, 151-742 South Korea; 33 National Laboratory Animal Center, National Applied Research Laboratories (NARLabs), 128 Yen-Chiou-Yuan Rd., Sec. 2, Nankang, Taipei, 11529 Taiwan; 34 IMPC, San Anselmo, CA 94960 USA; 35 Institute for Diabetes and Obesity, Helmholtz Diabetes Center at Helmholtz Zentrum München, German Research Center for Environmental Health (GmbH), 85764 Neuherberg, Germany; 36 Division of Metabolic Diseases, Department of Medicine, Technische Universität München, 80333 Munich, Germany; 37 Institute of Developmental Genetics, Helmholtz Zentrum München, German Research Center for Environmental Health GmbH, Ingolstädter Landstrasse 1, 85764 Neuherberg, Germany; 38 Chair of Developmental Genetics, Center of Life and Food Sciences Weihenstephan, Technische Universität München, Ingolstädter Landstrasse 1, 85764 Neuherberg, Germany; 39 Deutsches Institut für Neurodegenerative Erkrankungen (DZNE) Site Munich, Feodor-Lynen-Str. 17, 81377 Munich, Germany; 40 Munich Cluster for Systems Neurology (SyNergy), Adolf-Butenandt-Institut, Ludwig-Maximilians-Universität München, Feodor-Lynen-Str. 17, 81377 Munich, Germany; 41 Chair of Experimental Genetics, School of Life Science Weihenstephan, Technische Universität München, Alte Akademie 8, 85354 Freising, Germany; 42 Department of Internal Medicine, Division of Endocrinology, Diabetology, Vascular Medicine, Nephrology and Clinical Chemistry, University of Tübingen, 72076 Tübingen, Germany; 43 Institute for Diabetes Research and Metabolic Diseases of the Helmholtz Center Munich at the Eberhard-Karls-University of Tuebingen, Otfried-Müller-Str. 10, 72076 Tübingen, Germany; 44 Institute of Pharmaceutical Sciences, Department of Pharmacy and Biochemistry, Eberhard Karls University Tübingen, 72076 Tübingen, Germany; 45 Clinical Cooperation Group Type 2 Diabetes, Helmholtz Zentrum München and Ludwig-Maximilians Universität München, Ingolstädter Landstr. 1, 85764 Neuherberg, Germany; 46 Internal Medicine Nephrology and Center for Computational Medicine & Bioinformatics, University of Michigan, Ann Arbor, MI 48109 USA; 48 Institute of Pathology, Helmholtz Zentrum München, German Research Center for Environmental Health GmbH, Ingolstädter Landstrasse 1, 85764 Neuherberg, Germany; 49 Max-Delbrück-Centrum für Molekulare Medizin, Robert-Rössle-Str. 10, 13125 Berlin, Germany; 50 Berlin Institute of Health, Anna-Louisa-Karsch Str. 2, 10178 Berlin, Germany; 51 Helmholtz Zentrum Munich, Institute of Molecular Toxicology and Pharmacology, Ingolstädter Landstraße 1, 85764 Neuherberg, Germany; 52 Department of Infection and Immunity, Luxembourg Institute of Health, Esch-sur-Alzette, 4354 Luxembourg; 53 Department of Dermatology and Allergy Center, Odense Research Center for Anaphylaxis, University of Southern Denmark, 5000 Odense, Denmark; 54 Center of Allergy & Environment (ZAUM), Technische Universität München, and Helmholtz Zentrum München, Ingolstädter Landstrasse, 85764 Neuherberg, Germany; 55 Comprehensive Pneumology Center, Institute of Lung Biology and Disease, Helmholtz Zentrum München, German Research Center for Environmental Health (GmbH) and Member of the German Center for Lung Research, Ingolstädter Landstraße 1, 85764 Neuherberg, Germany; 56 Department of Neurology, Friedrich-Baur-Institut, Ludwig-Maximilians- Universität München, Ziemssenstrasse 1a, 80336 Munich, Germany; 57 German Center for Vertigo and Balance Disorders, University Hospital Munich, Campus Grosshadern, Marchioninistraße 15, 81377 Munich, Germany; 58 Institute for Medical Microbiology, Immunology and Hygiene, Technical University of Munich, Trogerstrasse 30, 81675 Munich, Germany; 59 Department of Cardiology, University of Heidelberg, Im Neuenheimer Feld 410, 69120 Heidelberg, Germany; 60 Institute of Molecular Psychiatry, Medical Faculty, University of Bonn, Sigmund-Freud-Strasse 25, 53127 Bonn, Germany; 61 Queen Mary University of London, London, E1 2AD UK; 62 Department of Molecular Physiology and Biophysics, Baylor College of Medicine, Houston, TX 77030 USA; 63 Charles River Laboratories, Wilmington, MA 01887 USA; 64 Korea Research Institute of Bioscience and Biotechnology, 30 Yeongudanji-ro, Ochang-eup, Cheongwon-gu, Cheongju-si, Chungcheongbuk-do 28116 Korea; 65 National Cancer Center, 323 Ilsan-ro, Ilsandong-gu, Koyang, 410-769 Korea

## Abstract

Metabolic diseases are a worldwide problem but the underlying genetic factors and their relevance to metabolic disease remain incompletely understood. Genome-wide research is needed to characterize so-far unannotated mammalian metabolic genes. Here, we generate and analyze metabolic phenotypic data of 2016 knockout mouse strains under the aegis of the International Mouse Phenotyping Consortium (IMPC) and find 974 gene knockouts with strong metabolic phenotypes. 429 of those had no previous link to metabolism and 51 genes remain functionally completely unannotated. We compared human orthologues of these uncharacterized genes in five GWAS consortia and indeed 23 candidate genes are associated with metabolic disease. We further identify common regulatory elements in promoters of candidate genes. As each regulatory element is composed of several transcription factor binding sites, our data reveal an extensive metabolic phenotype-associated network of co-regulated genes. Our systematic mouse phenotype analysis thus paves the way for full functional annotation of the genome.

## Abstract (web summary)

The genetic basis of metabolic diseases is incompletely understood. Here, by high-throughput phenotyping of 2,016 knockout mouse strains, Rozman and colleagues identify candidate metabolic genes, many of which are associated with unexplored regulatory gene networks and metabolic traits in human GWAS.

## Introduction

Metabolic disorders, including obesity and type 2 diabetes mellitus, are major challenges for public health. High initial treatment costs are compounded by complications that arise after diagnosis, including a number of consequential diseases, which together generate a significant burden for health care systems^1–5^. Genetic variations play established roles in the susceptibility and pathogenesis of these diseases^6–11^. However, identification of the underlying gene variants and their pathogenic roles is difficult because (1) functional annotation is still not available for many genes, especially genes that may be involved in disease but currently lack biological characterization^12^. (2) It also became clear that gene variants do not work in isolation but as parts of networks^13^. (3) There are concerns regarding the reproducibility, predictability, and relevance of results obtained from genotype–phenotype associations in disease model organisms^14^.

At the International Mouse Phenotyping Consortium (IMPC), we are generating a comprehensive catalog of mammalian gene functions to gain functional insights for every protein-coding gene, by producing and phenotyping more than 20,000 knockout mouse strains^15,16^. Knockout strains are analyzed in a comprehensive, standardized phenotyping screen that covers multiple areas of biology and disease with 14 compulsory test procedures and several additional optional tests (wwww.mousephenotype.org/impress). Phenotyping is conducted in 10 research centers in Europe, North America, and Asia. Each site follows the IMPC’s standardized operating procedures (IMPReSS), which were developed during the pilot programs EUMORPHIA and EUMODIC^17,18^. Standardization, data quality control, an automated statistical analysis pipeline, and the phenotyping of reference strains to assess inter-center variation all help to ensure robust and reproducible data^19–22^. Adherence to each of these standards enables derivation of a high-quality, powerful hypothesis-generating resource that includes genes from a substantial proportion of the entire mouse genome. Here we analyze phenotyping data of 2016 knockout strains for strong metabolic phenotypes and identify 974 known and previously unannotated genes with relevance for metabolic diseases. Twenty-three genes for which we find new strong metabolic phenotypes also have links to human metabolic disorders. We introduce a focus on network/context analysis to demonstrate how systemic phenotype information can be linked to known metabolic pathways. This pathway mapping revealed an unexpected degree of metabolic dimorphism between sexes. In addition, phenotype-associated regulatory networks allow the prediction of previously unknown gene functions. Therefore, our results underline the value of the IMPC resource for gene function discovery and augment the translational potential of metabolic phenotypes in mice.

## Results

### Strong metabolic phenotypes in IMPC mutants

**Fig. 1:**
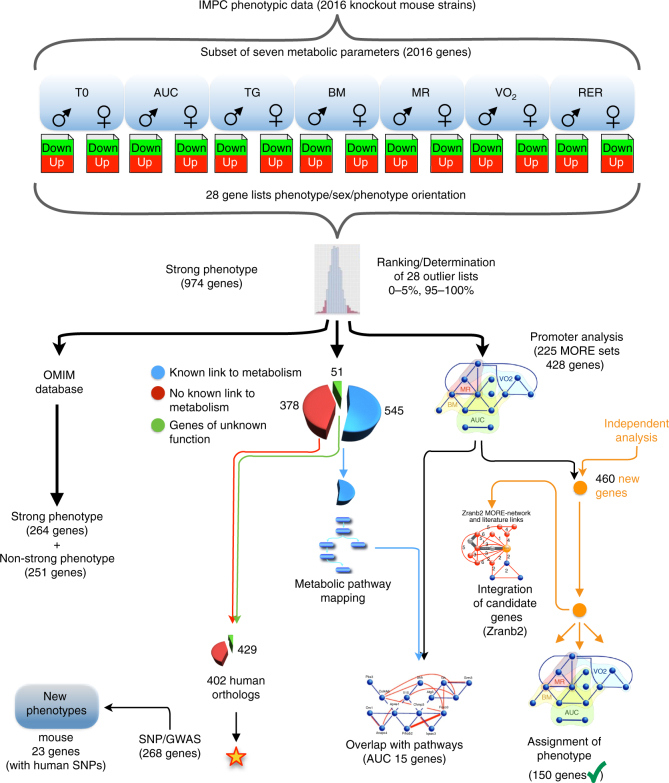
Strategical abstract depicting the research strategy to identify new genetic elements in metabolism. The IMPC phenotyping data of 2016 knockout mouse strains was systematically evaluated for new links to human metabolic disorders. Nine hundred seventy-four knockout strains showed a strong metabolic phenotype. This set of genes was used as data mining resource. In a multiple line of evidence approach, we finally identified 23 genes that were linked to human disease-related SNPs

**Fig. 2:**
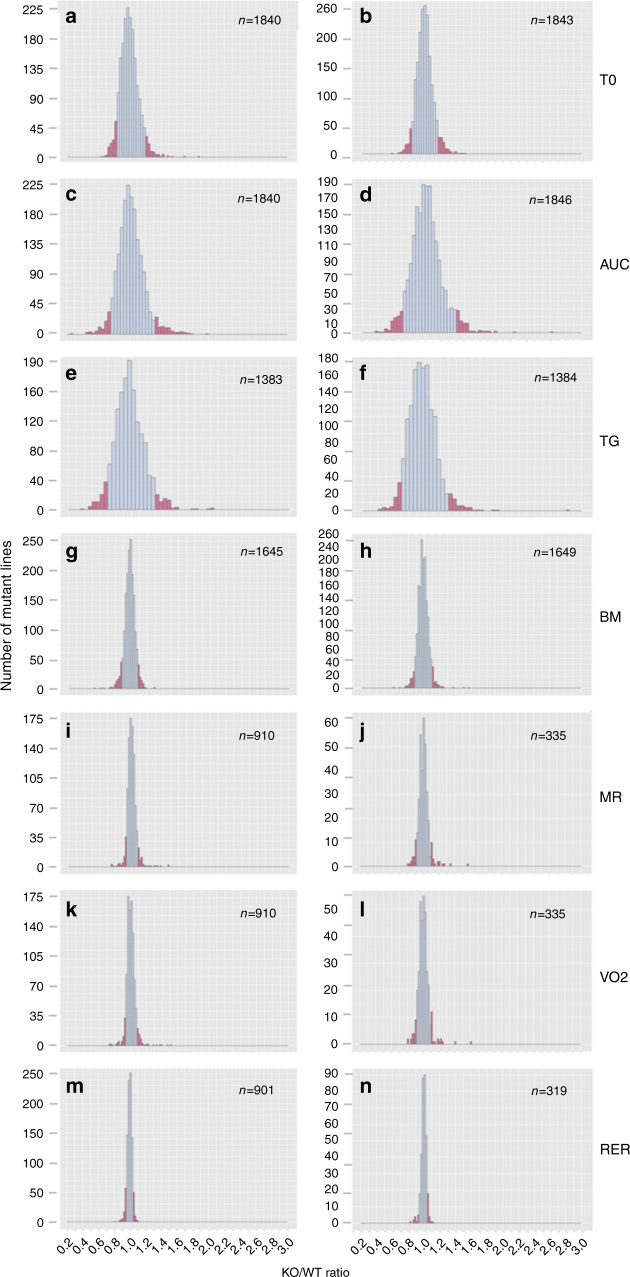
Frequency distribution of mutant/wild-type ratios for metabolic parameters, separated for males and females. **a** T0 females, basal blood glucose after overnight food deprivation, **b** T0 males, basal blood glucose after overnight food deprivation, **c** AUC females, area under the curve of blood glucose excursions after glucose injection in a glucose tolerance test, **d** AUC females, area under the curve of blood glucose excursions after glucose injection in a glucose tolerance test, **e** TG females, plasma triglyceride concentrations, **f** TG males, plasma triglyceride concentrations, **g** body mass females, **h** body mass males, **i** MR females, metabolic rate obtained from a 21 h indirect calorimetry trial, **j** MR females, metabolic rate obtained from a 21 h indirect calorimetry trial, **k** VO_2_ females, oxygen consumption obtained from a 21 h indirect calorimetry trial, **l** VO_2_ males, oxygen consumption obtained from a 21 h indirect calorimetry trial, **m** RER females, respiratory exchange ratio, **n** RER females, respiratory exchange ratio. Filled areas of the distributions cover the <5% and >95% strong metabolic phenotype genes, *n* provides number of mutant lines

We analyzed a total of 2016 IMPC mouse strains that were homozygous for a single-gene knockout on a C57BL/6N background or heterozygous when homozygotes were lethal or sub-viable (see Fig. 1 for a study overview). We chose seven metabolic parameters with diagnostic relevance in human clinical research for our study: fasting basal blood glucose level before glucose tolerance test (T0), area under the curve of blood glucose level after intraperitoneal glucose administration relative to basal blood glucose level (AUC), plasma triglyceride levels (TG), body mass (BM), metabolic rate (MR), oxygen consumption rate (VO_2_), and respiratory exchange ratio (RER), which is a measure of whole-body metabolic fuel utilization. To identify the universal cross-project metabolic characteristics of knockouts, we calculated mean mutant/wild-type ratios separately for all contributing centers^23^. Males and females were analyzed separately because sexual dimorphism is common in disease-related phenotypes^24,25^ (Fig. 1, top part). For all parameters, mutant/wild-type ratios were distributed around a modal value of 1.00, which would be expected when mutant and wild-type mice on average did not differ in the respective parameter (Table 1 and Fig. 2a–n). The shape of the metabolism phenotype mutant/wild-type ratio distributions differed between some parameters more than others. Triglycerides, basal blood glucose, and glucose clearance varied more between mutant and wild-type mice than RER, BM, VO_2_, and MR. For further analysis, we focused on gene knockout strains with a strong metabolic phenotype and compiled lists of genes for which the knockout resulted in mutant/wild-type ratios below the 5th percentile and above the 95th percentile of the ratio distributions (shown as the filled areas in Fig. 2). Based on these thresholds, we generated 28 gene lists, one for every sex parameter combination. Our 28 lists included a total of 974 “strong metabolic phenotype” genes (Fig. 1, second part from top). We used these genes as a data mining resource for further investigation into potential links to human metabolic disorders (see Supplementary Data 1—Mutant wildtype ratios for complete gene lists and Supplementary Data 2—Strong metabolic phenotype genes for strong phenotype genes).

**Table 1:** Number of genes analyzed and candidate hits identified by phenotyping mutant and wild-type mice from both sexes

Parameter	Number of genes			Females and males (in brackets: expected number if sex equally affected)		
	Females	Males	Both	Outlier <5%	Outlier >95%	Outlier <5% and >95%
T0	1843	1840	1839	162 (96)	166 (96)	324 (192)
AUC 0–120	1846	1840	1839	172 (96)	163 (96)	334 (192)
Triglycerides	1384	1383	1380	124 (73)	129 (72)	249 (145)
Body mass	1649	1645	1645	121 (79)	143 (86)	264 (165)
Metabolic rate	335	910	329	55 (48)	53 (48)	108 (96)
VO_2_	335	910	329	58 (48)	52 (46)	110 (94)
RER	319	901	313	55 (47)	54 (46)	109 (93)
Total	1995	2012	2016	575	587	974

### Evaluation of false discovery rates

The reproducibility and robustness of large-scale biology projects depends on minimizing the risks of false-positive or false-negative results. Using a post hoc approach to evaluate the risk of false-negative findings, we compiled a list of 666 mouse and human genes with published links to obesity and type 2 diabetes^25–29^. We then used these candidate genes to estimate the rate of false-negative discoveries in our project (see Supplementary Data 3—Candidate genes for false negative discovery). Hundred and one knockout mutants of these genes had been phenotyped by the IMPC at the time when we conducted our analysis. Hundred of 101 had a detectable metabolic phenotype: 58 (57.4%) scored as “strong metabolic phenotype genes” (*p* value 0.04, null hypothesis was defined as no enrichment of “strong metabolic phenotype” genes, see “Methods” section for description of the permutation simulation). With only one exception (*Serpinf1*, 210 MGI: 108080), knockout mice of all other 43 remaining genes even though not scoring as “strong metabolic phenotype genes” had phenotypic deviations in metabolic parameters in the range of low 20th or high 80th percentiles of their respective frequency distributions (*p* value 0.047).

### New knockout mouse models for metabolic diseases

For 58 of our 666 candidate genes with known links to metabolic functions, the link was based only on GWAS data with no further evidence available. Based on a systematic search of the mouse genome informatics (MGI) curated database, knockout mouse models had not yet been described for 4 of these 58 candidate genes before our study: *Lypla1*^28^, *Rfx6*^26^, *Slc6a14*, and *Slc6a3*^29^. Knockout mouse models had been generated previously for another 15 candidate genes but these had either no proven link to metabolism or were not reported to have been tested for metabolic parameters. Our phenotype data can therefore identify new genetic disease mouse models that allow in-depth investigation of disease mechanisms and fill the gap between genome-wide association studies and functional validation in a mammalian model organism.

### Illuminating unexplored mouse metabolic genes

**Fig. 3:**
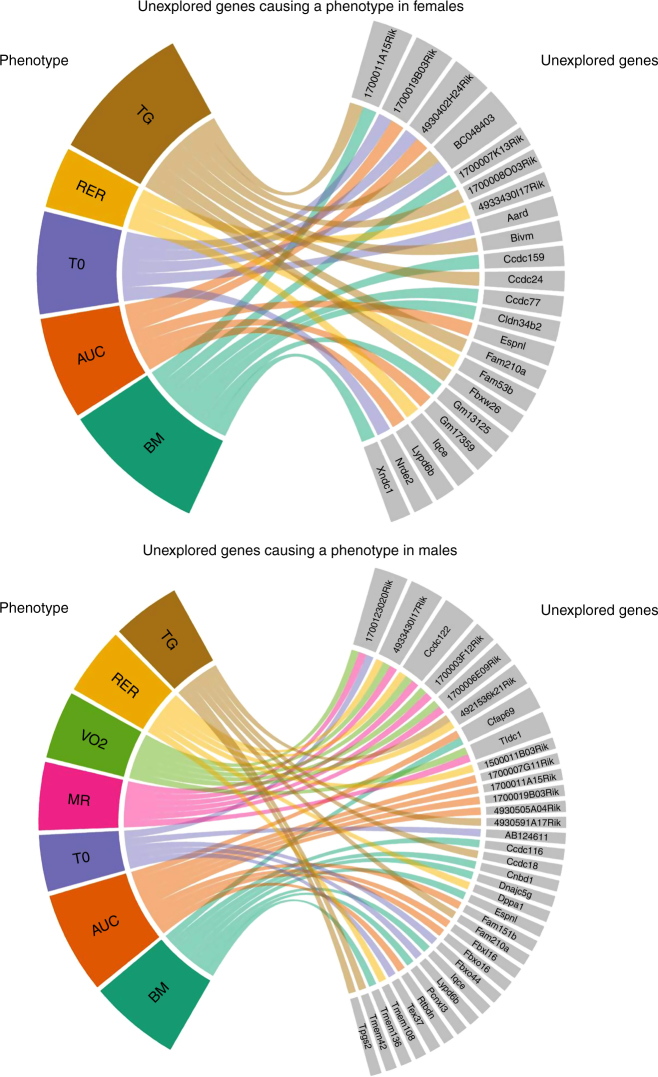
Links between unexplored metabolic genes and parameters that contribute to strong metabolic phenotypes in females (upper) and males (lower)

Many genes still lack any functional annotation. We conducted a systematic search of the MGI curated database to identify murine genes in our “strong metabolic phenotype” list with no previously known links to metabolism (see Supplementary Fig. 1 and Supplementary Data 4—Search results for unexplored metabolic genes). By careful stepwise evaluation, we found 429 of the 974 strong metabolic phenotype genes had no link to metabolic functions in mice. Fifty-one of these genes had no functional annotation at all (Fig. 1, center part). Their knockout caused strong phenotypes regarding glucose and energy metabolism (Fig. 3). Our data analysis provides evidence for new links to metabolic functions for these hitherto uncharacterized genes.

### Function of new metabolism genes is linked to human disease

We were interested in whether the strong metabolic phenotype genes could be mapped to diseases in the OMIM database. Of all 515 genes that were linked to at least one OMIM disease, 264 (27.1%) belonged to the strong metabolic phenotype genes, whereas 251 (24.1%) were “non-strong metabolic phenotype” genes (Fig. 1, lower left branch; also see Supplementary Data 5—OMIM mapping table).

**Fig. 4:**
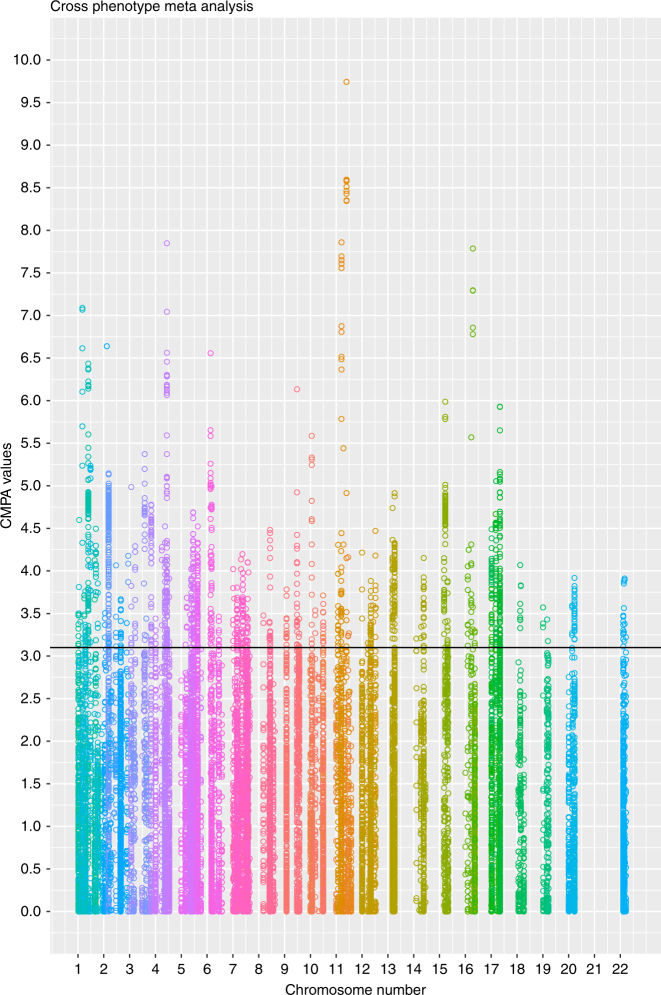
Cross phenotype meta-analysis of murine genes without prior link to metabolism. SNPs are on the *x*-axis ordered as per chromosome and the CPMA values (log transferred) are on the *y*-axis. The chromosomes are shown in different colors. Each column represents the genes and SNPs stacking vertically. The higher the CPMA measure, the higher the significance of SNPs across different phenotypes. SNPs above CPMA = 3.1 were considered to have a significant link to the disease traits

As a next step, we searched for non-annotated disease links to connect previously unknown strong metabolic phenotype genes to human disease. We found human orthologues for 402 of the 429 strong metabolic phenotype mouse genes. Note these 429 genes had no previously described link to metabolism and that includes the 51 completely unexplored mouse genes and 378 genes, which had no link to metabolism so far. Next, we extracted single-nucleotide polymorphisms (SNPs) in the SNiPA database^30^, from a ±2 kb region around the 402 human orthologues of strong phenotype genes (Fig. 1, second lower branch from the left). The SNiPA database has both functional annotations and linkage disequilibrium information for bi-allelic genomic variants (SNPs and single-nucleotide variations), based on the 1000 Genome Project. We found 19,253 SNPs for 268 of the 402 orthologous genes. SNiPA SNPs have various annotation layers such as gene annotations, associated phenotypic traits, and expression based quantitative trait loci. For each SNP, we evaluated the extent of association across 16 type 2 diabetes-related traits using the cross phenotype meta-analysis method on data from the DIAGRAM, MAGIC, GIANT, GLGC, and ICBP GWAS consortia^30–40^ (Fig. 4). We applied different levels of evidence and *p* value thresholds to infer association with disease traits (Table 2). By applying a standard genome-wide significance level of *p* ≤ 5 × 10^−8^, we found SNPs in 17 gene regions (*MTNR1B, MTCH2, SLC39A8, NUTF2, PABPC4, DNAJC5G, TCF19, PACSIN3, EVI5, EPB41L4B, DMXL2, RPTOR, CCDC18, RPGRIP1L, PCNXL3, WNT3, ELMO3*) that were associated with human metabolic phenotypes. By applying a significance level of 2.6 × 10^−6^ and correcting for all the SNPs we looked up in GWAS data, we found that more SNPs in *CCDC18* and in four additional genes (*CFAP69, IQCE, LYPD6B*, and *NRDE2*) were strongly linked to human phenotypes (Fig. 4). Regarding the remaining genes, all SNPs with a *p* value above 0.05 nevertheless had weak links to metabolic phenotypes in humans and only seven of the 268 genes with SNPs (*CMTM5, PPP1R14A, DUSP5, FGFBP3, TIMM22, WNT6,* and *PPP1R35*) lacked potential links to metabolic phenotypes.

**Table 2:** Translation to human disorders

*p* value—class across metabolic phenotypes in human GWAS data	SNPs	Genes	Unexplored metabolic genes
>5.0e−08	89	17	3
2.59e−06 to 5.0e−08	52	12	1
1.0e−03 to 2.59e−06	1071	94	5
0.05 to 1.0e−03	9107	254	20
>0.05	8912	233(7)	17(0)

Search for single-nucleotide polymorphisms in prioritized genes from IMPC in a cohort of pre-diabetic patients

The number of genes in the category >0.05 not overlapping with genes from other categories is displayed in brackets

As a next step, we corroborated the role of so-far unexplored mouse metabolic genes in a well-phenotyped clinical cohort. We analyzed the associations of common SNPs (i.e., those with minor allele frequencies ≥0.05) in the human orthologues of these murine genes with five human metabolic phenotypes: body fat content/distribution, blood glucose, insulin sensitivity, insulin secretion, and plasma lipids. To do this, we assimilated genome-wide genotype data, generated with Illumina’s Infinium^®^ Global Screening Array, from 2788 participants of the Tübingen Family (TÜF) study for type 2 diabetes^41^. We identified 240 common, bi-allelic, and non-linked SNPs (call rates ≥0.75) in 37 orthologues and analyzed them in the additive inheritance model by multiple linear regression analysis. Potential confounders were accounted for, e.g., gender, age, BMI, and insulin sensitivity, where appropriate. After Bonferroni correction for the number of SNPs tested, we found two SNPs below the study-wide significance threshold of *p* < 0.000213. These are the minor allele of SNP rs11734172 in *C4orf22*, which is associated with reduced insulin sensitivity (HOMA-IR, *p* = 8.8 × 10^−5^; ISI Matsuda, *p* = 6.2 × 10^−5^), and the minor allele of SNP rs76378941 in *CNBD1* which is associated with reduced plasma triglycerides (*p* = 0.00020). We identified seven additional non-linked SNPs in *C4orf22* and two additional non-linked SNPs in *CNBD1* with nominal associations (*p* < 0.05) with insulin sensitivity and triglycerides, respectively (Fig. 1, bottom left; all association results are shown in Supplementary Data 6—Association results of the TÜF study). To address whether the identified SNPs in *C4orf22* and *CNBD1* likely affect other nearby genes, we explored linkage disequilibrium data of the CEU population (99 Utah residents of Central European origin) from phase 3 of the 1000 Genomes Project (http://grch37.ensembl.org/Homo_sapiens/Info/Index). Neither the *C4orf22* nor the *CNBD1* gene locus had any adjacent disequilibrium. Thus, the identified SNPs in *C4orf22* and *CNBD1* likely do not affect nearby genes. So in conclusion, our single-gene based analysis identified a total of 23 orthologous genes with a link to human disease traits. We next focused on context and network aspects of our findings.

### Pronounced metabolic sexual dimorphism on gene level

As we had discovered connections between several strong metabolic phenotype murine genes and human disease, we next focused on the biological gene networks that underlie these phenotypes and their interconnections. First, we mapped our strong metabolic phenotype genes to metabolic pathways in the KEGG repository^42^. At least one knockout gene for each metabolic phenotype analyzed mapped to a metabolic pathway (males and females, (Supplementary Data 7—Metabolic pathways in KEGG). We present three interesting findings: (i) genes from different pathways caused comparable metabolic phenotypes in males and females (Supplementary Data 8–14—Pathway maps). (ii) The 13 male and 13 female strong metabolic phenotype genes for triglyceride levels that were linked to the global metabolic pathway map of the KEGG database had one gene in common, *Hpse*. However, 13 male and 13 female strong metabolic phenotype genes mapped to the same pathway (out of 121 male and 121 female genes). (iii) Some metabolic pathways were only found in the set of female strong metabolic phenotype genes, and not in males affecting plasma triglyceride levels. These pathways were “glycerophospholipid metabolism,” “linoleic acid metabolism,” and “ether lipid metabolism” pathways. We mapped the strong triglyceride-phenotype genes to non-metabolic biological pathways in the KEGG database, this also revealed sex-specific differences (Supplementary Data 7—Metabolic pathways in KEGG). These results give insight into and confirm sexual dimorphism in genes and pathways associated with metabolic phenotypes in mice.

### Regulatory networks of strong metabolic phenotype genes

Metabolic pathways require simultaneous and coordinated presence of functionally connected proteins. Therefore, we analyzed next whether our strong metabolic phenotype genes have common molecular regulatory features within their promoters. Transcriptional co-regulation often involves a common set of transcription factor-binding sites shared between co-regulated promoters. This concept of higher-level organized promoter wiring is known as multiple organized regulatory element (MORE) cassettes (see Supplementary Note 1). First, we extracted the promoter sequences of all our strong metabolic phenotype genes from the mouse genome sequence and analyzed them for the presence of identical MORE cassettes. We required promoter sets to contain at least three promoters. Analysis of all promotor sets for shared MORE cassettes resulted in a total of 225 sets of one or more MORE cassettes. These MORE sets were associated with at least one of the four sub-phenotypes (male, female, up or down). They were subdivided into these four subtypes by sex, up or down (top and bottom percentiles, respectively) of the seven metabolic phenotypes, e.g., AUC female low or VO_2_ female high. These associated MORE sets were present in 428 promoters of unique genes (Fig. 1, central part right). Genes sharing the same MORE set in at least one of their promoters thus belong to one regulatory network. We obtained a grouping of the collection of knockout genes independent of prior knowledge by the regulatory promotor analysis.

**Fig. 5:**
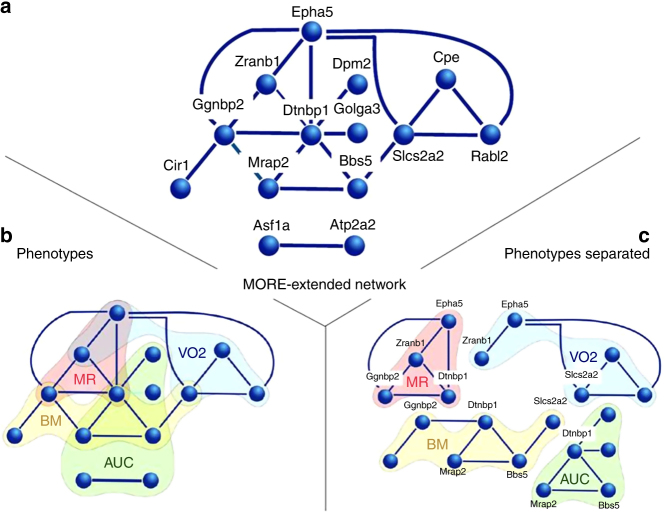
MORE set-derived network of the 20 genes having two or more phenotypic associations. Genes were selected from the strong metabolic phenotype list with two or more phenotypic associations to metabolic traits. Genes were chosen as targets to examine shared links to regulatory networks. **a** Connection of 12 genes and 2 other genes by MORE sets found in their promoters. **b** The MORE-derived network from **a** with gene–phenotype associations superimposed as colored areas. This superposition joins the 14 genes to one network. **c** Four phenotypes are shown separated from each other along with the links between these strong metabolic phenotype genes. This will facilitate recognition of the individual phenotypes

We next selected genes from our strong metabolic phenotype list that have two or more phenotypic associations with glucose homeostasis (18/20), body mass regulation (10/20), metabolic rate (2/20), and substrate utilization (2/20). The list of genes selected was *Asf1a*, *Atp2a2, Bbs5*, *Cir1, Commd9*, *Cpe*, *Dpm2*, *Dtnbp1*, *Epha5*, *Ggnbp2*, *Golga3, Il31ra*, *Lbp, Mboat7, Mrap2, Rabl2, Scrib*, *Slc2a2*, *Zranb1*, and *Zfpl1* (see Supplementary Data 15—Genes with metabolic phenotype). We chose these 20 genes as targets to examine shared links to regulatory network links. These links were defined by MORE cassettes and MORE sets. Overall, we found several MORE sets in corresponding promoters and the majority of the MORE sets were in more than one gene promoter in the 20 genes set (Table 3). This overlap allowed us to construct an association network with 14 of the 20 genes (Fig. 5). Interestingly, 12 of these genes were present in one single network indicating potentially coordinated expression of these genes (Fig. 5a). The overrepresented MORE sets thus connect genes within a specific phenotype, which suggests that individual regulatory mechanisms are confined to phenotypic subtypes (Fig. 5b). The overlap between different phenotypes is mainly due to genes that have more than one associated phenotype (Fig. 5c). Twelve of the 20 examined genes have common regulatory structures in their promoters that link them to a network. The edges of the network in Fig. 5c are shared MORE cassettes between the connected nodes (genes). Each edge is a phenotype or sub-phenotype association of the connected genes (nodes). Since MORE sets are molecular regulatory mechanisms, common MORE sets that link phenotype genes to each other indicate potential common regulatory mechanisms for the linked genes.

**Table 3:** Transcription factor-binding site alignment of MORE sets comprising a regulatory network of 14 genes

MORE cassette set														
AUC-mh MHS	dtnpb1							golga3						
AUC-mh PZE									asfa1	atp2a2				
AUC-mh XSSh	dtnpb1					bbs5								
AUC-ml PSH	dtnpb1										dpm2			
BM-fh CFS	dtnpb1		ggnbp2	slcs2a2		bbs5						mrap2		
BM-mh GEgE			ggnbp2										cir1	
MR-mh PBLS	dtnpb1		ggnbp2		zranb1									
MR-ml ASF	dtnpb1	epha5												
RER-mh XEE		epha5	ggnbp2											
RER-mh GSO		epha5					rabl2							
RER-ml MHS				slcs2a2		bbs5								
TG-fh XEgEg	dtnpb1		ggnbp2											
TG-fl NSF		epha5	ggnbp2											
VO2-mh XXCS		epha5			zranb1									
VO2-mh XXLSS		epha5		slcs2a2										
VO2-fh XCHS		epha5			zranb1									
VO2-fl XSSSf				slcs2a2			rabl2							cpe

### MORE sets and KEGG pathway mappings are overlapping networks

**Fig. 6:**
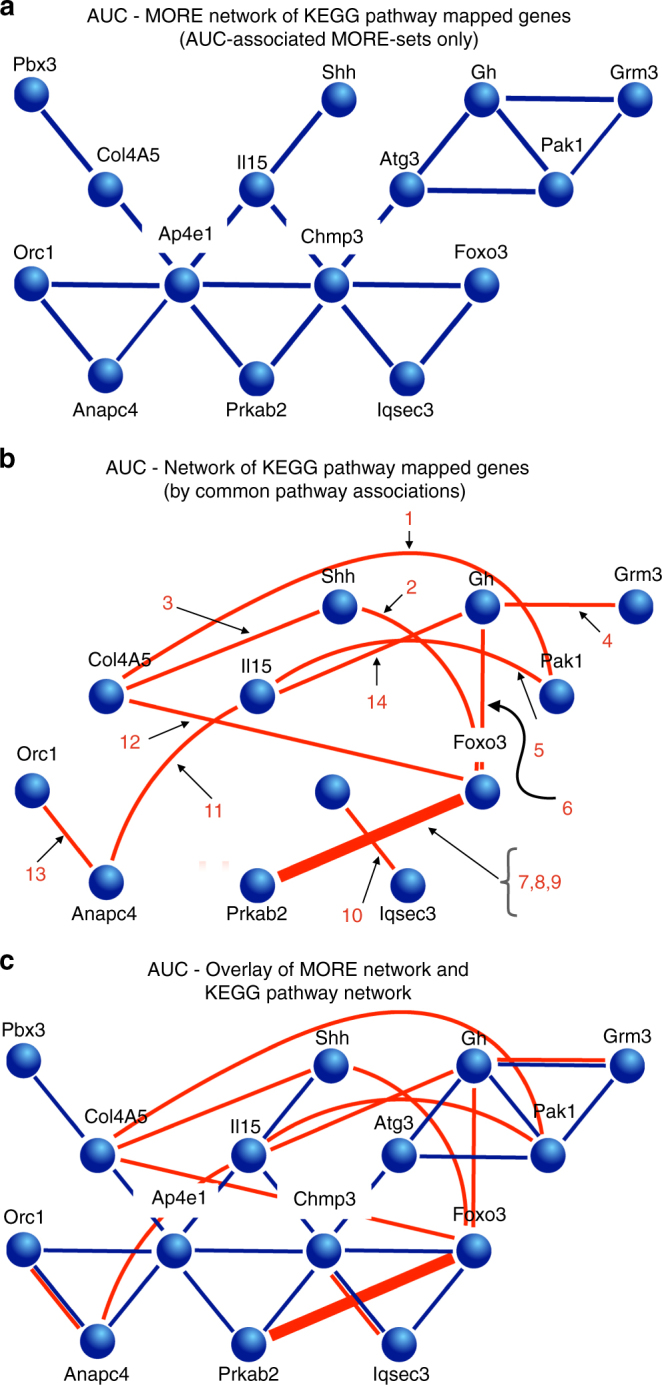
The joint MORE set and KEGG pathway network. Fifteen genes from the gene list AUC female impaired (KEGG) for which MORE set connections were also found. Male impaired did not yield results. **a** AUC gene network derived from KEGG pathway-mapped genes showing AUC-associated MORE sets only. **b** AUC gene network of KEGG pathway-mapped strong knockout genes by common pathway associations. **c** AUC overlay of MORE network and KEGG pathway network. (Legend extension: 1 = Focal adhesion—*Mus musculus* (mouse), 2 = JAK-STAT signaling pathway—*Mus musculus* (mouse), 3 = Pathways in cancer—*Mus musculus* (mouse), 4 = Neuroactive ligand-receptor interaction—*Mus musculus* (mouse), 5 = Chemokine signaling pathway—*Mus musculus* (mouse), 6 = PI3K-AKT signaling pathway—*Mus musculus* (mouse), 7 = FoxO signaling pathway—*Mus musculus* (mouse), 8 = AMPK signaling pathway—*Mus musculus* (mouse), 9 = Longevity regulating pathway—*Mus musculus* (mouse), 10 = Endocytosis—*Mus musculus* (mouse), 11 = HTLV-I infection—*Mus musculus* (mouse), 12 = PI3K-AKT signaling pathway—*Mus musculus* (mouse), 13 = Cell cycle—*Mus musculus* (mouse), 14 = Basal cell carcinoma—*Mus musculus* (mouse))

MORE sets are solely derived from promoter analysis without the use of prior knowledge of the phenotypes or the genes involved. Therefore, the resulting networks are independent of knowledge, often exceeding current knowledge. Because of that, validation of the regulatory network connection by existing current knowledge is rather limited. However, the genes within these regulatory networks are well-known and many are already associated with known pathways. We thus selected the genes that mapped to KEGG pathways and that had a strong glucose clearance phenotype to construct a MORE set network of 15 genes (Fig. 1, third lower branch from the left and Fig. 6a). A network for those 15 genes was constructed in which the edges were KEGG pathway networks to which both connected genes (nodes) mapped (Fig. 6b). Importantly, the two networks could be superposed, which thus validates combining our data-driven MORE set approach with experimental knowledge-based KEGG pathway mapping (Fig. 6c). In summary, our analysis supports the concept that the various initially unconnected knockout genes form well-organized functional metabolic networks. These networks are supported at several system levels (phenotype, regulation, and metabolism).

### Prediction of metabolic genes

We hypothesized that the presence of MORE cassettes in currently unannotated genes could be used to functionally characterize them. Therefore, we proceeded determining which of the promoter sequences of the ~22,000 protein-coding genes in the mouse genome matched to the MORE cassettes defined in this study. We analyzed promoters of an independent set of genes that was not part of the original data set used to define the initial MORE cassettes (original data set: IMPC Release 4.2 from Dec 2015; additional set of genes: IMPC Release 4.3 from Apr 2016). The complementary data set contained 757 genes that had completed IMPC phenotyping after the earlier Release 4.2. A batch query of phenotype terms, from the IMPC portal (www.mousephenotype.org), for these genes showed that 150 of 460 genes (32.6%) with 1–11 MORE cassette matches had a metabolic phenotype, whereas only 68 of the total 297 genes (22.9%) with no MORE cassette matches had such a metabolic phenotype (32.6% vs. 22.9%, *p* = 0.004, Fisher’s exact test; Fig. 1, right lower branch). Thus, the presence of phenotype-associated MORE cassettes does indeed predict genes with a corresponding phenotype.

**Fig. 7:**
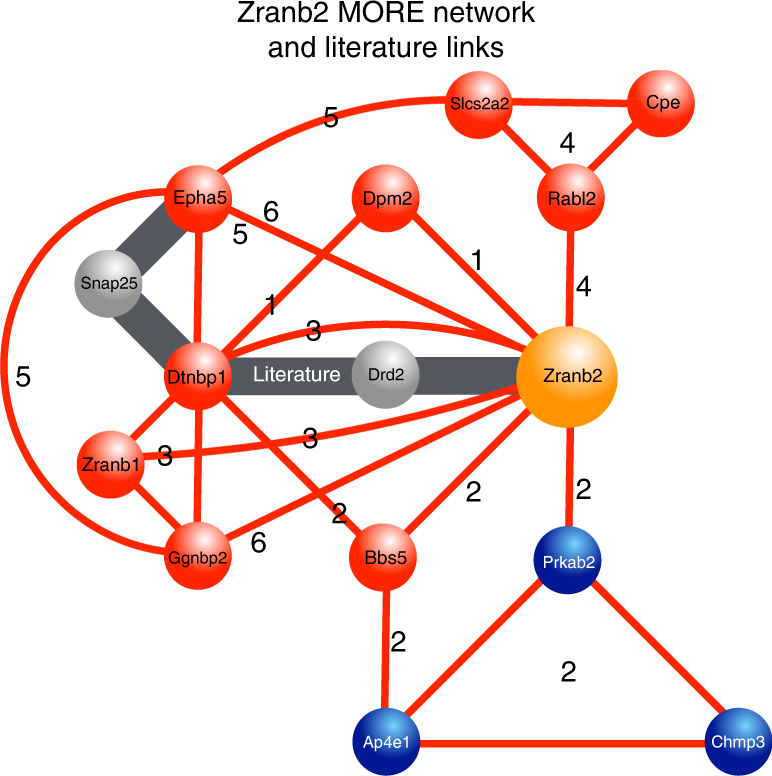
*Zranb2* MORE network. Validation of predicted gene functions based on six shared MORE sets and functional links from literature (further explanation see text)

The 9 genes with 6–11 MORE cassette matches associated with metabolic phenotypes were linked to the MORE set network defined in this study. This analysis also provided additional support for the relative position of the gene in the network in several cases. Figure 7 shows the *Zranb2* gene, which has six MORE sets in its promoters. *Zranb2* shares two central MORE sets with the *Dtnbp1* and *Epha5* genes, which confirms their previously reported functional links with *Zranb2*^42–45^. Gauging the presence of phenotype-associated MORE cassettes in promoters is thus currently the only method to predict whether an uncharacterized gene has a candidate phenotype, or whether disruption of the gene will cause the respective phenotype (Fig. 1, second from right lower branch).

## Discussion

In this study, we have established and evaluated analysis and visualization tools that identify and select candidate genes with roles in glucose and energy metabolism starting from the pool of IMPC phenotype data of mouse knockout strains, which covers ~10% of mammalian protein-coding genes. This data set links new genes to disease-relevant metabolic phenotypes. As with all large-scale approaches, false discovery could be a major issue. We used 101 genes with a known or hypothesized link to obesity or type 2 diabetes to assess the false-negative discovery rate and our approach missed only one of the 101 genes. Therefore, metabolic phenotypes can be detected in almost all IMPC mouse models of published candidate genes for obesity and type 2 diabetes.

The pioneering systematic approach of our IMPC database is an advance toward the final frontier of genome functional annotation as 44% of the strong metabolic phenotype genes have not been linked to metabolism in mice (429 of the 974). Furthermore, 51 of these new metabolism genes had no previous functional annotation at all. Strikingly, orthologues of 23 of these genes had single-nucleotide polymorphisms associated with human metabolic disease phenotypes. With large-scale projects like the IMPC, adding functional annotation and disease links to so-far unannotated genes will continuously contribute to closing the systems biology knowledge gap in all fields, e.g., for unexplored metabolic genes. The data analysis approaches we describe are based on snapshot computational evaluation of current knowledge that is changing fast. Since our initial analysis, new suggested links to physiological functions of three so-far unexplored genes (*AB124611, Dppa1,* and *GM13125*) were found in an updated literature search.

The problem of candidate genes without established links to existing knowledge is as “old” as high-throughput analysis itself. These “out of context” genes are very hard to characterize functionally and pure statistical association (e.g., gene ontology analysis) is insufficient to understand their biological context. Since it is well established that genes only work in collaboration with other genes, our analysis focuses on elucidating the genetic context of newly discovered candidate genes. Our approach allows targeted verification of a simple “guilt-by-association” hypothesis that infers that a gene likely contributes to a specified pathway if it is frequently found in our lists alongside genes with a common function.

Our findings of wide-spread pathway and network structures underlying individual gene findings provide a new integrative view to our results. Our pathway network approach pinpointed genes that caused specific and interrelated metabolic phenotypes in specific genetic backgrounds. For example, we confirmed recently published findings regarding a strong sexual dimorphism with minimal or no shared genes between males and females with the same associated phenotypes^46^. The metabolic discordance between males and females was generally lower at the pathway level than at the gene level. For example, only 1 out of 13 genes was common between the 13 strong metabolic phenotype genes for triglyceride levels for each sex, whereas both sets of 13 genes mapped to the same pathways.

Our pathway mapping necessarily used prior knowledge, like the metabolic (KEGG) pathways, which cannot be derived from newly inputted experimental data. We complemented this prior knowledge-based approach by a purely data-driven (prior knowledge-independent) analysis using MORE sets. This MORE set analysis deciphered the molecular regulatory networks in the promoters of the strong metabolic phenotype genes. Our approach therefore detects functionally connected gene networks that are supported by several techniques.

Data-driven analysis has another important feature. MORE cassettes are invariant features like reading frames. It is therefore very likely that the promoters of some uncharacterized genes with the same phenotypes will have phenotype-associated MORE cassettes. If this is the case, it should be possible to use the presence of MORE cassettes for a priori prediction of phenotypes. We validated this hypothesis based on a set of newly phenotyped genes that were not part of our initial analysis. A considerable number of these genes also had metabolic phenotype-associated MORE sets. We found a significant correlation between the presence of these MORE sets and the link of genes to metabolic functions. Of course, also genes newly predicted based on MORE sets would be expected to link into the known context of genes for this phenotype. Therefore, we analyzed one example of a de novo predicted potential phenotype-causing gene (*Zranb2*) in more detail. We not only found additional verification for the pre-defined network, but also additional links in the literature supporting the network association of this gene. Therefore, the presence of characterized MORE sets provides opportunities for experimental planning of verification experiments.

In conclusion, we show here that our new multiple-line-of-evidence functional discovery approach, which is based on the IMPC phenotyping program, can identify new disease genes related to energy metabolism and glucose homeostasis. Our platform will thus enable researchers to prioritize research on so-far uncharacterized genes to fill the gap in functional annotation for metabolic genes. We predicted gene functions in a set of 757 subsequently phenotyped genes, based on the integration of functionally uncharacterized genes into established regulatory networks and functional contexts. By linking gene functions to metabolic disorders, our protocol and the identification of metabolic relevant genetic elements will accelerate the understanding of human disease.

## Methods

### Mouse husbandry and phenotyping

We used the IMPC data resource to identify genes associated with strong metabolic phenotypes in mice. The IMPC phenotyping pipeline includes 14 mandatory and several optional tests that cover all major disease areas (www.mousephenotype.org/impress). Phenotyping procedures are being conducted in 10 centers (Baylor College of Medicine BCM, Helmholtz-Zentrum München HMGU, PHENOMIN Institut Clinique de la Souris ICS, Jackson Laboratory JAX, Medical Research Council Harwell MRC Harwell, MARC Nanjing University NING, RIKEN BioResource Center RBRC, Toronto Centre for Phenogenomics TCP, Mouse Biology Program University of California Davis UC Davis, and Wellcome Trust Sanger Institute WTSI) in Europe, North America, and Asia (for details, see http://www.mousephenotype.org/about-impc/impc-members). Mice were generated on a C57BL/6N background and phenotyping data were collected between the age of 4 and 16 weeks following approved animal ethics protocols in every institution (see Supplementary Table 3 for license numbers).

### Phenotyping parameters

From the 509 phenotyping parameters assessed by the IMPC early adult phenotyping screen (http://www.mousephenotype.org/impress), we chose seven parameters: (1) basal blood glucose levels after overnight food deprivation (T0) and (2) the area under the curve (AUC) of glucose excursions during the intraperitoneal glucose tolerance test as a read out for glucose-stimulated insulin secretion and insulin sensitivity (IPGTT) [IMPC_IPG_001]; (3) non-fasted triglyceride levels from clinical chemistry [IMPC_CBC_003]; (4) body weight [IMPC_DEXA_001]; (5) metabolic rate (MR) normalized to body mass (see below); (6) oxygen consumption (VO_2_) normalized to body mass (see below); and (7) respiratory exchange ratio (RER = VCO_2_/VO_2_) from the indirect calorimetry trial [IMPC_CAL_003].

### Bioinformatics

In general, only unique genes were included, i.e., in the case that data for two different zygosities were available, we selected homozygotes instead of heterozygotes. We included lines in which data of males and females with the same genotype was available instead of having different genotypes for either males or females. Multiple entries for the same gene only occurred in reference strains. For each test procedure (indirect calorimetry, clinical chemistry, and glucose tolerance test), we received a csv file containing phenotyping data (one row per mouse, including characteristics such as sex, center, etc., parameters and metadata in columns). We conducted careful quality-control checks on each of the selected seven parameters and excluded obviously invalid values, which was only the case for VO_2_ and metabolic rate.

### VO_2_ and metabolic rate parameters

For VO_2_ and metabolic rate parameters, we used the body weight-independent residuals since body mass is the major determinant for variability in absolute VO_2_ and metabolic rate. For this, we calculated a linear model with mean body mass (mean individual mass before and after the calorimetry test) as predictor and VO_2_, respectively, metabolic rate as response variable separate for each phenotyping center and for each sex. The residuals of these models represent the difference between each individual’s actual VO_2_/metabolic rate and the response value predicted by their mean body weight. By adding a constant (e.g., the predicted value for the mean body weight) to the residual, the sense of an actual VO_2_/metabolic rate value is conveyed. The new residual response variable is finally uncorrelated with the mean body weight^47^.

### General statistics

The number of mutant strains assessed for each parameter varied depending upon (1) center-wide test implementation; and (2) whether both males and females were phenotyped; e.g., indirect calorimetry providing VO_2_, MR, and RER was not conducted in every center and not always using both sexes. The statistical power of the phenotyping approach implemented by the IMPC alliance was evaluated by data obtained from the pilot program EUMODIC^21^. Here a sample size of seven mutant mice per sex was found to be required to detect genotype effects. This is the outcome of a trade-off between sufficient statistical power and technical and workflow constraints, which are linked in any high-throughput phenotyping screen. As described above mean mutant/wild-type ratios were calculated for all parameters split for phenotyping center and sex to identify universal cross-project metabolic characteristics of knockouts. No further quantitative statistical measure was assigned to these pragmatic but restrictive selection criteria. However, critical values for the 5% tails could be computed by upper/lower threshold = mean ± 1.645 s.d., approximating a significance value of *p* < 0.05 for each tail or *p* < 0.1 for both higher and lower tails together.

### Permutation analysis

We performed permutation tests in order to estimate *p* values for the fraction of strong metabolic phenotype genes within the set of 101 IMPC genes with documented links to obesity and type 2 diabetes. In particular, we shuffled the phenotype ratios of the gene set analyzed by IMPC one million times and then we obtained sample distributions of the fraction of genes within the <5th >95th and the <20th and >80th range percentiles of the 101 genes in these random sets. These distributions were subsequently used to obtain the *p* values.

### Identification of unexplored genes

Starting with all genes with strong metabolic phenotype of the IMPC project (IMPC Release 4.2 from Dec 2015), several filters were applied to discard genes with known molecular or functional annotation. First, we removed genes linked to mammalian phenotype (MP) terms related to metabolism (as described in the next section “Mapping to MP ontology terms”). Next, genes with annotated information in KEGG or gene ontology (GO) databases were removed following the procedure described in “Representation of genes in KEGG graphical pathways” and “Selection of GO terms describing metabolic processes” Method sections, respectively. Literature information on the function of the resulting list of genes was inspected using PubMed, LitInspector^48^, and GeneCards^49^. This iterative process produced a list of 51 genes with no information. The additional 20 genes represent those genes with known molecular information (based on GO molecular properties) but unknown biological processes (See Supplement Fig. 1).

### Representation of genes in KEGG graphical pathways

The Interactive Pathways Explorer v2 web-based tool^50^ was used to visualize the strong metabolic phenotype genes on a map representing global metabolism in mice. To that aim, we mapped the strong metabolic phenotype and “non-strong” metabolic phenotype genes for each parameter and sex to the global “Metabolic pathways” overview map from *Mus musculus* organism constructed using 146 KEGG pathways.

To get an overview of the representation of the strong metabolic phenotype genes of each parameter and sex in biological pathways, we mapped the genes to the graphical KEGG pathways provided by KEGG online website. In these pathway maps, several functionally related genes might be grouped in the same node. When strong metabolic phenotype and “normal” genes of the same parameter and sex mapped to the same group, we highlighted the strong metabolic phenotype genes.

To link genes to KEGG pathways, the KEGG website search tool (http://www.genome.jp/kegg/tool/map_pathway1.html) was used applying the filter “organism: *Mus musculus*.” The information available from the page was then downloaded and processed using Bash shell and R scripts (R version 3.3.2). To identify those genes linked to metabolism, we selected those mapping to the “Metabolism” class of KEGG classification.

### Selection of GO terms describing metabolic processes

Associations of genes to GO terms were extracted from Gene Ontology Consortium website^51^. We analyzed terms of the “Molecular function” and “Biological processes” domains. To select genes involved in metabolism based on GO term annotation, we selected all GO terms under the “metabolic process” category and mapped them to the genes of interest.

### Mapping to MP ontology terms

MP terms for the genes of interest were extracted from the MGI database (http://www.informatics.jax.org/) using the file (MGI_PhenoGenoMP.rpt), which contains information on genes and their annotated phenotypes. In order to avoid circular discoveries, as the MGI database includes the IMPC phenotype data, IMPC entries were removed using internal filters.

### Human subjects, GWAS, and SNP analysis and statistics

Orthologous/paralogous genes of mouse metabolism genes mapping to the human genome were used for analysis. We searched for SNPs in a ±2 kb region in SNiPA database. For each SNP occurring in or around genes, we evaluated the extent of this sharing for 19238 SNPs in 16 metabolic phenotype GWAs from various consortia, including DIAGRAM, MAGIC, GIANT, GLGC, and ICBP^30–36^. All participants of the studies contributing to the consortia have given their informed consent for genetic studies, which was confirmed by the appropriate ethics committees. For our present study, we used metadata without any link to individual IDs or data only. We used cross phenotype meta-analysis (CPMA), which detects association of a SNP to multiple, but not necessarily all, phenotypes^37^. The CPMA analysis applies the likelihood ratio test that measures the likelihood of the null hypothesis (i.e., that the significant SNP is uniformly distributed across consortiums) over the alternative hypothesis.

The ongoing Tübingen family (TÜF) study for type 2 diabetes currently comprises more than 3000 unrelated non-diabetic Caucasian individuals at increased risk of type 2 diabetes (subjects with family history of diabetes, BMI ≥27, impaired fasting glycemia, and/or previous gestational diabetes). The participants were comprehensively characterized by anthropometrics, five-point oral glucose tolerance tests, and clinical chemistry^41^ and were genotyped on Illumina’s Infinium^®^ Global Screening Array-24 v1.0 BeadChip, which was developed based on Phase-III data of the 1000 Genomes Project and which has 700,078 single-nucleotide polymorphisms (SNPs). The study followed the principles laid down in the Declaration of Helsinki and was approved by the Ethics Committee of the University of Tübingen. Informed written consent was obtained from all participants. From the TÜF study, we selected 2788 subjects with complete phenotypic data sets (body fat content/distribution, blood glucose, insulin sensitivity, insulin secretion, and plasma lipids) as the study population for the analysis of the human orthologues of unexplored murine metabolic genes. Of these 51 murine genes, four (*1500011B03Rik*, *4930591A17Rik*, *Dppa1*, and *Cldn34b2*) had no human orthologues, and three orthologues (*PRAMEF20*, *C17orf105*, and *TMEM42*) were not on the array. In the remaining 44 orthologues, only SNPs with minor allele frequencies ≥0.05 were analyzed, due to statistical power limitations of the study population. In consequence, seven orthologues (*C5orf52*, *CCDC24*, *CCDC116*, *FBXW12*, *TMEM136*, *DNAJC5G*, and TEX37) with no common SNPs were excluded from the analyses. In the remaining 37 genes, 240 common, bi-allelic, and non-linked (*r* < 0.8) SNPs with call rates ≥0.75 were identified and ultimately analyzed. Analysis of association with the aforementioned phenotypes was carried out by multiple linear regression analysis (least squares method) to account for potential confounders (gender, age, BMI, and insulin sensitivity) whenever appropriate. The SNPs were analyzed in the additive inheritance model. According to Bonferroni correction for the number of SNPs tested in parallel, *p* values <0.000213 were considered significant. Associations were indicated as nominal if *p* values were ≥0.000213 and <0.05.

### MORE cassette enrichment analysis (promoter analysis)

Regarding promoter selection, sets of genes were collected from genes in the 5% outlier range (sex, high and low outliers were separately analyzed). The promoters of these gene sets were collected with the ElDorado database and the program Gene2Promoter (both Genomatix, Munich).

Multiple organized regulatory elements (MORE) form a (partial) “fingerprint” that is present within a group of regulatory regions (promoters, enhancers, etc.). Transcriptional MORE cassettes use transcription factor-binding sites (TFBSs) as elements, which are defined by a weight-matrix-based detection method (MatInspector, Genomatix, Munich).

A MORE cassette is defined by several individual TFBSs and corresponding detection thresholds, their order and strand orientation in the DNA sequence, and the distance ranges and distance variations between pairs of TFBS elements (See Supplementary Notes 1 and 2 for more details).

Since there are too many potential MORE cassettes to collect in a database for standard enrichment analysis (see Supplementary Note 1 for details), we determined the number and structures of the MORE cassettes actually present in at least three promoters of each set. Each promoter set was analyzed separately for the presence of MORE cassettes shared by at least three promoters per set using the program FrameWorker (Genomatix, Munich). Parameters were set according to the supplier’s defaults, except for Sequence quorum which was adapted top-down until MORE cassettes were found. The distance range variation was set to 20 bp, the minimum distance between elements was set to 10 and the minimum range of elements in MORE cassettes was set to 3–6. No further adjustments were made unless the minimum setting of three sequences did not reveal any MORE cassettes. In this case, the distance variation was increased to 30. Promoter sets that still had no MORE cassettes with at least three elements were considered negative.

To define MORE cassette sets following procedure was applied: In cases where multiple MORE cassettes were detected, a manual alignment of the MORE cassettes was carried out and all MORE cassettes that were identical in all elements and in their order were collected into a MORE cassette set (only varying in the distance definitions). In cases of four or more elements, MORE cassettes were also collected into one bin if they differed only in one element at the exact same relative position within the MORE (e.g., A-B-C-D and A-B-F-D), however, in such cases the overlap of the promoters harboring one of these MORE cassettes was required to exceed 80%. The resulting MORE cassette sets were treated as if they were individual MORE cassettes. However, detection in promoters by ModelInspector was always based on actual individual MORE cassettes.

All MORE cassettes and the respective sets were located in promoter sequences by the program ModelInspector (Genomatix, Munich) in the following promoter sets: the set initially used for the detection of these MORE cassettes (to detect additional matches missed in the detection process, e.g., located on the opposite strand), the promoter set derived from the opposite outlier group (plus or minus, respectively), the corresponding promoter sets from the other sex, and finally the set of all promoters from the mouse genome as provided in the ElDorado database.

To analyze enrichment of MORE cassettes, we applied following procedures. From the total number of promoters containing a particular MORE cassette or MORE cassette set, as determined above, an expectation value was determined for a random subset of the same size as the test set (e.g., the original promoter set) and an over-representation against an expected random draw of a subset of the size of the sub-phenotype (male, female, up or down) gene promoters, comparing the promoter set of interest to all of the mouse promoters (and the total number of MORE sets actually present in all promoters). Any over-representation ≥2 was regarded as indication of an association of the MORE cassette or MORE cassette set with the specific promoter set analyzed. Enrichment was also analyzed in the set of genes with the corresponding opposite phenotype as a control (e.g., for female low, female high was checked).

To determine regulatory connections between phenotypes, all promoters from all genes selected for all phenotypes were analyzed for presence of each enriched MORE cassette or MORE set as determined from that phenotype. This analysis was carried out across all phenotypes for both sexes and regardless of the high/low differentiation. Genes with any of the enriched MORE cassettes or MORE sets in at least one of their promoters were collected. In this case, enrichment with phenotypes other than the initial one from which the MORE cassettes were determined was not required.

The list of the 20 most interesting genes was checked for the presence of associated MORE cassettes in their promoters. Then a network was constructed in which genes associated with the same MORE sets in the same sub-phenotype (male, female, up or down) were “connected” via this shared MORE structure in their promoters. The resulting network was superimposed onto closed areas that represent the respective phenotype, the MORE sets or the genes they were associated with.

### Ethical approval

All details regarding animal ethics approval of mouse production, breeding and phenotyping at each center are provided in Supplementary Table 3. All procedures were conducted in compliance with each center’s ethical animal care and use guidelines. All procedures were in accordance with the respective national legislation. In addition, we confirm that all efforts were made to minimize suffering by considerate housing and husbandry. Animal welfare was assessed routinely for all mice.

### Data availability

All phenotyping data and mouse lines presented in this paper are openly available from the IMPC portal and via our FTP site (ftp://ftp.ebi.ac.uk/pub/databases/impc/latest/). Information on strong metabolic phenotype gene, GWAS results and network analysis are provided in Supplementary Data files. The complete list of MORE cassette descriptions is available from the authors, consisting of the salient features: individual TFBS elements, their order, strand orientation, distance rage and distance range variations between the pairs. This allows location of the MORE cassettes, using any suitable TFBS program, without requiring access to the Genomatix Suite—for individual promoters. The list of MORE sets, consisting of the MORE cassettes belonging to each set is also available.

## Electronic supplementary material

Description of Additional Supplementary Files

Supplementary Information

Supplementary Data 1

Supplementary Data 2

Supplementary Data 3

Supplementary Data 4

Supplementary Data 5

Supplementary Data 6

Supplementary Data 7

Supplementary Data 8

Supplementary Data 9

Supplementary Data 10

Supplementary Data 11

Supplementary Data 12

Supplementary Data 13

Supplementary Data 14

Supplementary Data 15
