# Supplementary material for: Identification of genetic elements in metabolism by high-throughput mouse phenotyping
Source: Nat Commun. 2018 Jan 18;9:288. doi: 10.1038/s41467-017-01995-2 (PMC5773596; doi:10.1038/s41467-017-01995-2)
Supplement: Supplementary file 1 — Description of Additional Supplementary Files [file 41467_2017_1995_MOESM1_ESM.pdf]

Description of Additional Supplementary Files

File Name: Supplementary Data 1
Description: **Mutant/wildtype ratios of all 2,016 IMPC lines.** Information on phenotyping center, gene ID, and a yes/no tag for strong metabolic phenotype are provided.

File Name: Supplementary Data 2
Description: **List of strong metabolic phenotype genes.** Mutant/wildtype ratios for all 974 genes causing a strong metabolic phenotype genes.

File Name: Supplementary Data 3
Description: **List of candidate genes used for *post hoc* evaluation of false negative discovery.** Gene lists with candidate genes for type 2 diabetes and obesity were extracted from key publications in this area.

File Name: Supplementary Data 4

Description: List of so far unexplored metabolic genes detected by stepwise literature and database search for known functional links. The filtering search strategy is depicted in supplementary figure 1.

File Name: Supplementary Data 5
Description: **Mapping of OMIM disease links.** OMIM disease links were searched both for strong metabolic phenotype and genes not causing a strong metabolic phenotype.

File Name: Supplementary Data 6
Description: Association analyses of common SNPs in 37 unexplored genes with metabolic phenotypes in the TÜF study. Common (minor allele frequency ≥0.05), bi-allelic, and non-linked SNPs from Illumina's Infinium Global Screening Array were analyzed for association with body fat content/distribution, blood glucose, insulin sensitivity, insulin secretion, and plasma lipids by multiple linear regression analysis with gender, age, BMI, and insulin sensitivity as confounding covariates as indicated (a, b, c). The SNPs were analyzed in the additive inheritance model, and p-values and effect sizes (standardized beta) of the minor alleles are reported. According to Bonferroni correction for the number of SNPs tested in parallel, p-values <0.000213 were considered significant (cells marked by red shading). Associations were indicated as nominal if p-values were ≥0.000213 and <0.05 (cells marked by yellow shading).

File Name: Supplementary Data 7
Description: **Mapping of IMPC strong and normal phenotype genes to metabolic pathways in KEGG.**

File Name: Supplementary Data 8
Description: **Mapping of genes strongly affecting basal blood glucose (T0) to the KEGG global "Metabolic pathway".** Genes affecting basal blood glucose were mapped to the KEGG global "Metabolic pathway" (web-based tool: Interactive Pathways Explorer v21). Black, red, and blue lines represent genes showing a strong metabolic phenotype only in males, only in females, or both in males and females, respectively. Grey lines represent genes with non-strong metabolic phenotype. A line in the map denotes groups of functionally related genes. Conflicts arising by the mapping of genes with strong and non-strong metabolic phenotypes to the same line were resolved by selecting always the gene with a strong metabolic phenotype.

File Name: Supplementary Data 9
Description: **Mapping of genes strongly affecting glucose clearance (AUC) to the KEGG global "Metabolic pathway".** Genes affecting the area under curve of glucose excursion during a glucose tolerance test were mapped to the KEGG global "Metabolic pathway" (web-based tool: Interactive Pathways Explorer v21). Black, red, and blue lines represent genes showing a strong metabolic phenotype only in males, only in females, or both in males and females, respectively. Grey lines represent genes with non-strong metabolic phenotype. A line in the map denotes groups of functionally related genes. Conflicts arising by the mapping of genes with strong and non-strong metabolic phenotypes to the same line were resolved by selecting always the gene with a strong metabolic phenotype.

File Name: Supplementary Data 10
Description: **Mapping of genes strongly affecting triglycerides (TG) to the KEGG global "Metabolic pathway".** Genes affecting plasma triglyceride levels were mapped to the KEGG global "Metabolic pathway" (web-based tool: Interactive Pathways Explorer v21). Black, red, and blue lines represent genes showing a strong metabolic phenotype only in males, only in females, or both in males and females, respectively. Grey lines represent genes with non-strong metabolic phenotype. A line in the map denotes groups of functionally related genes. Conflicts arising by the mapping of genes with strong and non-strong metabolic phenotypes to the same line were resolved by selecting always the gene with a strong metabolic phenotype.

File Name: Supplementary Data 11
Description: **Mapping of genes strongly affecting body mass (BM) to the KEGG global "Metabolic pathway".** Genes affecting body mass were mapped to the KEGG global "Metabolic pathway" (web-based tool: Interactive Pathways Explorer v21). Black, red, and blue lines represent genes showing a strong metabolic phenotype only in males, only in females, or both in males and females, respectively. Grey lines represent genes with non-strong metabolic phenotype. A line in the map denotes groups of functionally related genes. Conflicts arising by the mapping of genes with strong and non-strong metabolic phenotypes

to the same line were resolved by selecting always the gene with a strong metabolic phenotype.

File Name: Supplementary Data 12
Description: **Mapping of genes strongly affecting metabolic rate (MR) to the KEGG global "Metabolic pathway".** Genes affecting metabolic rate were mapped to the KEGG global "Metabolic pathway" (web-based tool: Interactive Pathways Explorer v21). Black, red, and blue lines represent genes showing a strong metabolic phenotype only in males, only in females, or both in males and females, respectively. Grey lines represent genes with non-strong metabolic phenotype. A line in the map denotes groups of functionally related genes. Conflicts arising by the mapping of genes with strong and non-strong metabolic phenotypes to the same line were resolved by selecting always the gene with a strong metabolic phenotype.

File Name: Supplementary Data 13
Description: **Mapping of genes strongly affecting oxygen consumption (VO2) to the KEGG global "Metabolic pathway".** Genes affecting oxygen consumption were mapped to the KEGG global "Metabolic pathway" (web-based tool: Interactive Pathways Explorer v21). Black, red, and blue lines represent genes showing a strong metabolic phenotype only in males, only in females, or both in males and females, respectively. Grey lines represent genes with non-strong metabolic phenotype. A line in the map denotes groups of functionally related genes. Conflicts arising by the mapping of genes with strong and non-strong metabolic phenotypes to the same line were resolved by selecting always the gene with a strong metabolic phenotype.

File Name: Supplementary Data 14
Description: **Mapping of genes strongly affecting metabolic fuel utilization (RER) to the KEGG global "Metabolic pathway".** Genes affecting metabolic fuel utilization were mapped to the KEGG global "Metabolic pathway" (web-based tool: Interactive Pathways Explorer v21). Black, red, and blue lines represent genes showing a strong metabolic phenotype only in males, only in females, or both in males and females, respectively. Grey lines represent genes with non-strong metabolic phenotype. A line in the map denotes groups of functionally related genes. Conflicts arising by the mapping of genes with strong and non-strong metabolic phenotypes to the same line were resolved by selecting always the gene with a strong metabolic phenotype.

File Name: Supplementary Data 15
Description: **Information on prominent metabolic phenotypes of 20 genes that were used for constructing regulatory and metabolic networks.**
