# Supplementary material for: Identification of genetic elements in metabolism by high-throughput mouse phenotyping
Source: Nat Commun. 2018 Jan 18;9:288. doi: 10.1038/s41467-017-01995-2 (PMC5773596; doi:10.1038/s41467-017-01995-2)
Supplement: Supplementary file 2 — Supplementary Information [file 41467_2017_1995_MOESM2_ESM.doc]

**Supplementary Figure 1**


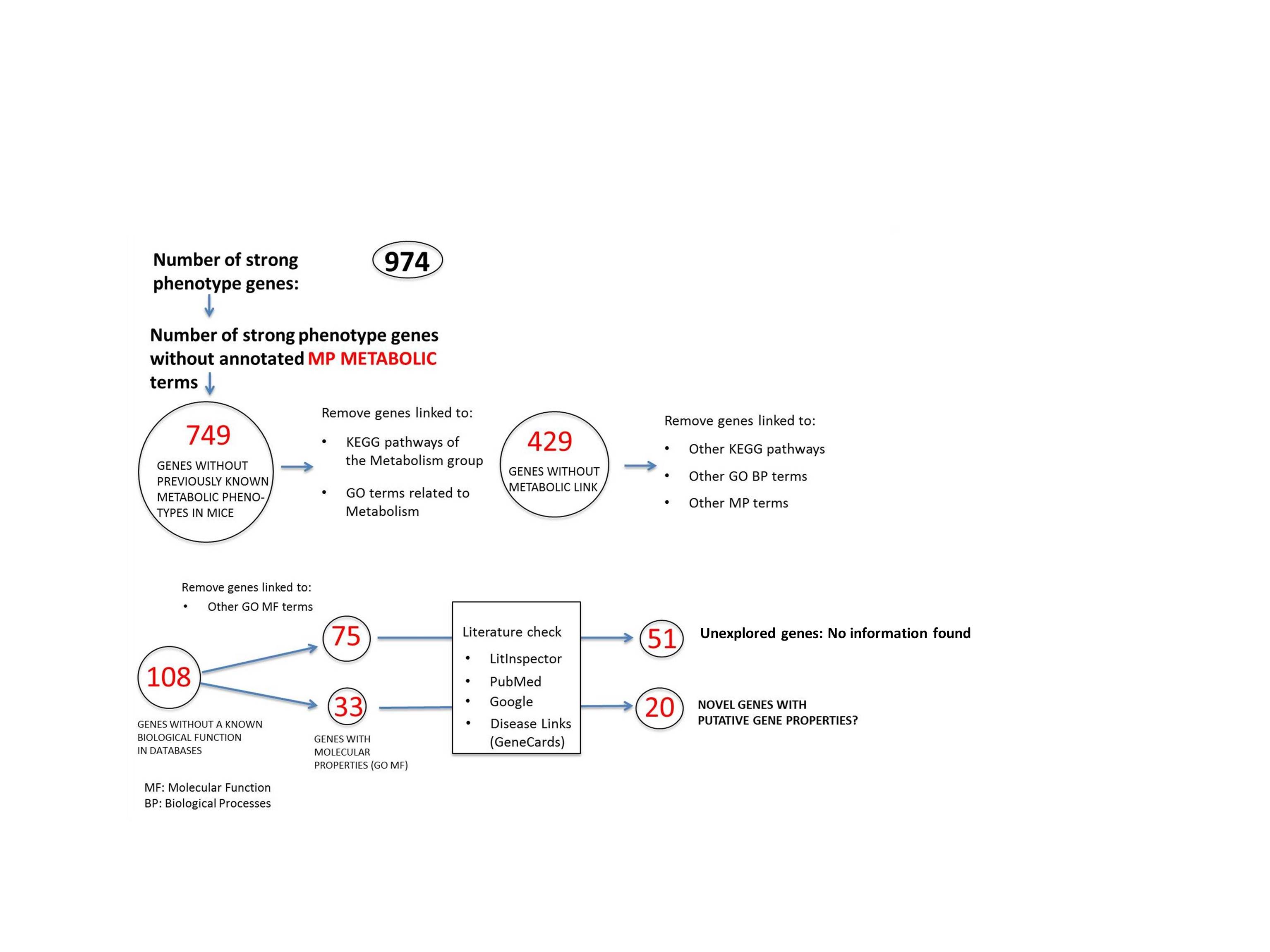


**Supplementary Figure 1:** Stepwise search strategy to discover so far unexplored genes. For 749 of the outlier genes no MP term linked to metabolic functions in mice could be detected. When removing all genes with reference to KEGG pathways and GO terms linked to metabolism, 429 genes remained with no previous annotation. We then filtered for other MP terms, KEGG pathway or GO terms resulting in 108 genes without a known biological function in databases. 75 of those had no link to a GO term category molecular functions. In a final step we conducted a thorough literature check with LitInspector, Pubmed, and Google. We could not detect any information for 51 genes.

**Supplementary Table 1: Selected GO-terms associated with the 34 predominant TFBSs in associated MORE-sets.**

|  | **GO-term** | **GO-term** | **Go-term** |
| --- | --- | --- | --- |
| AHRR | positive regulation of glycolytic process | positive regulation of vascular endothelial growth factor receptor signaling pathway | positive regulation vascular endothelial growth factor production |
| BEDF | - |  |  |
| CLOX | - |  |  |
| E2FF |  | cell proliferation | regulation of cell cycle |
| EGRF | response to glucose | positive regulation of cell proliferation | response to insulin |
| ETSF | cell differentiation | cell proliferation |  |
| FKHD | cell differentiation | cellular glucose homeostasis | vascular endothelial growth factor receptor signaling pathway |
| GCMF | - |  |  |
| GFI |  | cell proliferation |  |
| HAND | cell differentiation |  |  |
| HEAT | - |  |  |
| HNF6 |  |  | pancreas development |
| HOMF | cell differentiation | negative regulation of vascular endothelial growth factor receptor signaling pathway | cellular response to growth factor stimulus |
| HOXF | negative regulation of cell differentiation |  |  |
| HUB1 | - |  |  |
| IRXF | - |  |  |
| KLFS | regulation of cell differentiation |  | glucose transport |
| LHXF | - |  |  |
| MEF2 |  | cellular response to growth factor stimulus |  |
| MEF3 | - |  |  |
| MIZ1 |  |  | negative regulation of cell cycle |
| MOKF | - |  |  |
| MTEN | - |  |  |
| MYT1 | - |  |  |
| PCBE | - |  |  |
| PLAG |  |  | cell cycle arrest |
| SMAD | response to glucose | negative regulation of cell proliferation | insulin secretion |
| SORY | cell differentiation | response to growth factor | cell cycle arrest |
| SP1F |  | cell proliferation |  |
| STAT | growth hormone receptor signaling pathway | cell proliferation | cellular response to insulin stimulus |
| TAIP | - |  |  |
| VTBP | - |  |  |
| YBXF | - |  |  |
| ZICF | - |  |  |
|  |  |  |  |

**Supplementary Table 2: Green color indicates match of both phenotype and subtype, yellow indicates right phenotype but wrong subtype.**

| gene | phenotype MORE-sets | MORE-set | phenotypes from screen |
| --- | --- | --- | --- |
| Cpe | MR fl | LFSXTC | T0 h |
|  | VO2 fl | XSSSf | AUC h |
| Rabl2 | BM ml | GiEE | BM fh |
|  | RER mh | GSO | AUC |
|  | RER fh | XPZE | TG fh |
|  | VO2 fl | XSSSf | T0 h |
| Slc2a2 | BM fh | CFS | BM h |
|  | TG fl | XSSS | TG h |
|  | T0 fl | MXSS | T0 h |
|  | RER fh | XPZE | AUC fh |
|  | RER ml | MHS |  |
|  | VO2 fl | XSSSf |  |
|  | VO2 mh | XXLSS |  |
|  |  |  |  |
| Epha5 | T0 ml | DHS | T0 h |
|  |  |  | AUC h |
|  | TG fl | NSF | TG mh |
|  | TG fl | ASF |  |
|  | TG fl | PBS |  |
|  | TG fl | DHS |  |
|  | TG fl | DSX |  |
|  | RER fh | XPZE |  |
|  | RER mh | GSO |  |
|  | RER mh | XEE |  |
|  | BM fl | XSSSF |  |
|  | VO2 fh | XCHS |  |
|  | VO2 mh | XXLSS |  |
|  |  |  |  |
| Zranb1 | AUC mh | GEK |  |
|  | TO ml | DHS |  |
|  | T0 ml | SEgE |  |
|  | MR mh | HCFFSF |  |
|  | MR mh | PBLS |  |
|  | RER fh | XPZE |  |
|  | RER fh | PIKSZE |  |
|  | RER ml | EEk |  |
|  | BM fh | GEE |  |
|  | BM ml | SXE |  |
|  | BM ml | HEE |  |
|  | TG fl | PBS |  |
|  | TG fh | XEEg |  |
|  | TG ml | PBLS |  |
|  | VO2 fl | XSSSf |  |
|  | VO2 fh | PFSS |  |
|  | VO2 fh | XCHS |  |
|  | VO2 ml | BSSH |  |
|  | VO2 mh | XXLSS |  |
|  |  |  |  |
| Ggnbp2 | Metrate male high P10 | PBLS |  |
|  | RER male high P8 | NZP |  |
|  | RER male high P8 | XEE |  |
|  | BM female high P12 | CFS |  |
|  | BM female high P12 | PEE |  |
|  | BM female high P12 | NKK |  |
|  | BM male high P12 | GEgE |  |
|  | Triglyceride female low P11 | NSF |  |
|  | Triglyceride female high P11 | XEEg |  |
|  | Triglyceride female high P11 | XEgEg |  |
|  | Triglyceride male high P11 | DSX |  |
|  | Triglyceride male high P11 | HiEE |  |
|  | Triglyceride male high P11 | PXZE |  |
|  | VO2 male low P9 | GXXE |  |
| Dtnbp1 | AUC male low P4 | PSH |  |
|  | AUC male high P4 | MHS |  |
|  | AUC male high P4 | XSSSh |  |
|  | Metrate male low P10 | ASF |  |
|  | Metrate male high P10 | PBLS |  |
|  | RER female high P8 | XPZE |  |
|  | BM female high P12 | TEE |  |
|  | BM female high P12 | XZE |  |
|  | Triglyceride female low P11 | HHS |  |
|  | Triglyceride female high P11 | XEgEg |  |
|  | Triglyceride male high P11 | DSX |  |
|  |  |  |  |
| Golga3 | AUC male high P4 | MHS | AUC h |
|  | AUC male high P4 | MHS |  |
|  | AUC male high P4 | SZE |  |
|  | AUC male high P4 | SZE |  |
|  | T0 female low P5 | GSS |  |
|  | T0 female low P5 | GSS |  |
|  | T0 female high P5 | ASSS | T0 h |
|  | T0 female high P5 | ASSS |  |
|  | RER male high P8 | GNS |  |
|  | RER male high P8 | GNS |  |
|  | RER male high P8 | GSO |  |
|  | RER male high P8 | GSO |  |
|  | BM male high P12 | HEE |  |
|  | BM male high P12 | HEE |  |
| Dtnbp1 | AUC male low P4 | PSH |  |
|  | AUC male high P4 | MHS | AUC h |
|  | AUC male high P4 | XSSSh |  |
|  | Metrate male low P10 | ASF |  |
|  | Metrate male high P10 | PBLS |  |
|  | RER female high P8 | XPZE |  |
|  | BM female high P12 | TEE |  |
|  | BM female high P12 | XZE |  |
|  | Triglyceride female low P11 | HHS |  |
|  | Triglyceride female high P11 | XEgEg |  |
|  | Triglyceride male high P11 | DSX |  |
|  |  |  | T0 h |
| Dpm2 | AUC male low P4 | PSH |  |
|  | Triglyceride female low P11 | PBS |  |
| Cir1 | VO2 female low P9 | SSXN | BM h |
|  | VO2 female low P9 | XSSSf |  |
|  |  |  |  |
| Mrap2 | BM female high P12 | CFS | BM h |
|  | Triglyceride female high P11 | PSH | TG h |
|  | Triglyceride male high P11 | DSX |  |
|  | T0 female high P5 | ASSS | T0 h |
|  | AUC male high P4 | ZFSS | AUC h |
| Bbs5 | AUC female high P4 | BSSS | AUC h |
|  | AUC male high P4 | MHS |  |
|  | AUC male high P4 | HSPS |  |
|  | AUC male high P4 | XSSSh |  |
|  | BM female high P12 | CFS | BM h |
|  | Triglyceride female low P11 | AFS | TG h |
|  | Triglyceride female low P11 | PBS |  |
|  | VO2 female low P9 | XSSSf |  |
|  | VO2 female high P9 | XCHS |  |
|  | VO2 male low P9 | SXSD |  |
|  | VO2 male high P9 | XXLSS |  |
|  | T0 male low P5 | DHS | T0 h |
|  | RER male low P8 | MHS |  |

**Supplementary Table 3: Animal Ethics Approval Information**

| Institute | Information |
| --- | --- |
| BCM Baylor College of Medicine | Approval committee: Institutional Animal Care and Usage Committee. Approval License: AN-5896 |
| GMC Helmholtz Zentrum München | Approval committee: Regierung von Oberbayern. Approval License: 144-10 |
| ICS Mouse Clinical Institute | Approval Committee: Com'Eth N°17 and French Ministry for Superior Education and Research (MESR). Approval licenses: internal numbers 2012-009 & 2014-024. Approval licenses: MESR: APAFIS#4789-2016040511578546 |
| MRC Harwell | Approval committee:  Animal Welfare and Ethical review Board (AWERB). Approval License: 30/3384 |
| Nanjing University | Approval committee: IACUC of MARC. Approval License: NRCMM9 |
| RBRC RIKEN Tsukuba Institute, BioResource Center | Approval committee: The RIKEN Tsukuba Animal Experiments Committee. Approval License: Exp11-002, 12-002, 13-002, 14-002, 15-002, 16-002 Collection, maintenance, storage, breeding and distribution of the mouse resources Exp11-011, 12-011, 13-011, 14-009, 14-017, 15-009, 16-008 Phenotyping analyses and related studies in mice |
| The Centre for Phenogenomics | Approval committee: Animal Care Committee (ACC) of The Centre for Phenogenomics. Approval License: Animal Use Protocol (AUP) 0153, 0275, 0277, 0279 |
| The Jackson Laboratory | Approval: The Jackson Laboratory Institutional Animal Care and Use Committee (IACUC). License: NIH Office of Laboratory Animal Welfare (OLAW) assurance # D16-00170  Production Grant IACUC Protocol: 14004  Phenotyping Grant IACUC Protocol: 11005  Phenotyping Grant Supplement IACUC Protocol: 99066  Accreditation: AAALACi #000096 |
| UCD University of California, Davis | Approval committee: UC Davis Institutional Animal Care and Use Committee (IACUC). Approval License: Protocol #19075 |
| WTSI Wellcome Trust Sanger Institute | Approval committee: Animal Welfare and Ethical review Board (AWERB). Approval License: PPL 80/2076 Valid 27th Nov 2006 - 3rd Jan 2012; PPL 80/2485 valid 3rd Jan 2012 - 5th Dec 2016 |
| Institutes that breed the mice and collect phenotyping data are guided by their own ethical review panels and licensing and accrediting bodies, reflecting the national legislation under which they operate. Details of their ethical review bodies and licenses are provided here. All efforts were made to minimize suffering by considerate housing and husbandry. All phenotyping procedures were examined for potential refinements that were disseminated throughout the consortium. Animal welfare was assessed routinely for all mice involved. | |

**Supplementary Note 1: Introduction to Regulatory Networks - Definition and Properties of MORE-Cassettes**

Definition of MORE-cassette:

Multiple Organized Regulatory Elements forming a (partial) “fingerprint” present within a group of regulatory regions (this can describe all region composed of multiple elements with the only limitation that the nature of the elements needs to be known and a computerized method is available to locate them in sequences). Transcriptional MORE-cassettes use Transcription Factor Binding Sites (TFBSs) as elements and here a weight-matrix-based detection method (1, 2).

A MORE-cassette definition consists of several individual TFBSs & corresponding thresholds; their order & strand orientation; their distance ranges & distance variations (3, 4).


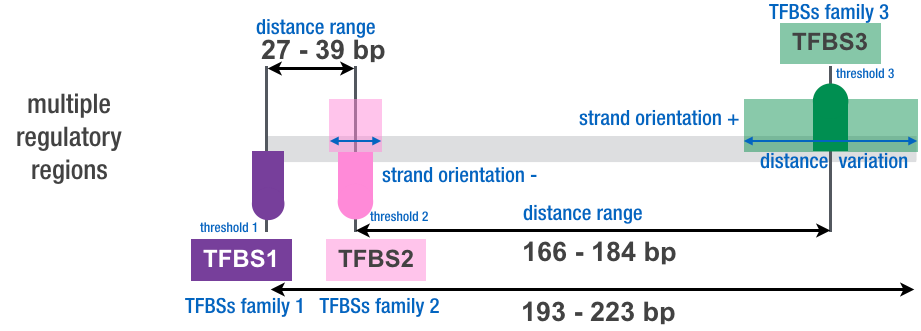


Properties of transcriptional MORE-cassettes:

Any TFBSs in a regulatory region can be part of multiple distinct MORE-cassettes.

Pars-pro-toto: A MORE-cassette present in m sequences can be found by analysis of any subset of n ≥ 3 of these sequences (with small variations especially in the distance range definition). The resulting MORE-cassette will find most if not all of the m sequences in subsequent searches (5-9).

Functional association: A MORE-cassette is only accepted as associated with a particular (transcriptional) function if it is associated with a set of sequences known to have this function vs all corresponding sequences in the genome (over-representation ≥ 2.0, e.g. a group of promoters vs a random selection of same size from all promoters in the genome).

Necessary / sufficient: A MORE-cassette is a necessary part of particular transcriptional functions, but not automatically sufficient for a particular function as the MORE-cassette could be incomplete (additional elements required for full and specific functionality).

Completeness: The subset can be complete or not. Maybe more elements are required; or additional features (enhancers, repeats, etc.) outside the MORE-cassette (e.g. for cell-specificity).

Function: The transcriptional function can be complete or not. A MORE-cassette characterizes a subset of sequences with a (partial) common function. The transcriptional function of MORE-cassettes is usually cell-type specific (10-12). The same MORE-cassette in the same promoter can be functionally irrelevant in another cell type.

Selectivity / specificity of transcriptional MORE-cassettes:

Selectivity: An incomplete MORE-cassette is not specific but selective. The over-representation vs. random pick is a measure of selectivity in this case. Unless there is evidence to the contrary all MORE-cassettes are regarded as incomplete by default.

Specificity: If a MORE-cassette is present only in regulatory regions of a particular transcriptional function, it is specific for this transcriptional function. This does not rule out other MORE-cassettes to be either associated with or also specific for the same transcriptional function. It does also not imply that the MORE-cassette is sufficient for the associated function.

If a MORE-cassette is unique in the genome and in a gene-associated region (e.g. sole match in a genome is in a particular promoter) then this MORE-cassette is specific for that gene (complete fingerprint).

If a MORE-cassette can be found in orthologous regions of other genomes (regardless if it is just selective or specific) then the MORE-cassette defines a phylogenetically conserved structure, but not necessarily a phylogenetically conserved function. This requires a functional association in addition (see properties), which is not necessarily phylogenetically conserved as well but could differ between species (13).

Occurrence of transcriptional MORE-cassettes

Combinatorics of MORE-cassettes: 209 TFBSs matrix families (MatBase 9.3), ≈ 100 TFBSs / promoter, 3 elements to one MORE-cassette result in possible combinations: (100*209)^3^ = 20900^3^ = 9.1*10^12^ / promoter in case of 5 elements this becomes: > 10^21^ / promoter. However, we currently have access to only ≈ 123,000 promoters (human, mouse ≈ 98,000, ElDorado 06/2015, NCBI build 38), which indicates that this combinatorial space will be virtually empty with respect to biologically existing combinations (similar situation as for protein folding).

Therefore, the enrichment starts not with an exhaustive database of potential MORE-cassettes but by determining which MORE-cassettes are actually present in a set of promoters (≥3) MORE-cassettes of 2 elements occur frequently in regulatory regions and often are involved in a transcriptional function but almost always require additional features, rendering them not selective enough to define any preferred location. They should only be considered in case of clear experimental proof of functionality (e.g. as in the module library). MORE-cassettes ≥ 3 elements are predominantly found in promoters (primary location), enhancers, and other regulatory regions. MORE-cassettes of ≥ 5 elements often are function- or even gene-specific. Gene-specific MORE-cassettes cannot be associated with a particular function of that gene without specific experimental evidence since individual genes often have several transcriptional functions and such MORE-cassettes could be composites of more than one MORE-cassette with different functions.

Evidence for regulatory functions of transcriptional MORE-cassettes:

TF-centered evidence: TF-binding and function studies (EMSA, footprint, TF-suppression/over-expression): readout: transcription/RNA.

MORE/TFBSs-centered evidence: modular interdependencies (Module-Library), phylogenetic conservation (partial, species-specific MOREs), SNPs altering MORE-TFBSs with allele-specific differences in RNA expression, targeted TFBS deletions/mutations of MORE-TFBSs resulting in the predicted signaling and transcriptional changes.

**Supplementary Note 2: Transscription Factor Binding Sites**

SORY mouse TFS:

HBP1 (ElDorado EntrezGene), HMGA1 (ElDorado EntrezGene),

HMGA2 (ElDorado EntrezGene), PINX1 (ElDorado EntrezGene),

SOX10 (ElDorado EntrezGene), SOX11 (ElDorado EntrezGene),

SOX13 (ElDorado EntrezGene), SOX17 (ElDorado EntrezGene),

SOX18 (ElDorado EntrezGene), SOX2 (ElDorado EntrezGene),

SOX21 (ElDorado EntrezGene), SOX3 (ElDorado EntrezGene),

SOX30 (ElDorado EntrezGene), SOX5 (ElDorado EntrezGene),

SOX6 (ElDorado EntrezGene), SOX7 (ElDorado EntrezGene),

SOX8 (ElDorado EntrezGene), SOX9 (ElDorado EntrezGene),

TOX (ElDorado EntrezGene)

FKHD mouse TFs:

Foxa1 (ElDorado EntrezGene), Foxa2 (ElDorado EntrezGene),

Foxa3 (ElDorado EntrezGene), Foxb1 (ElDorado EntrezGene),

Foxb2 (ElDorado EntrezGene), Foxc1 (ElDorado EntrezGene),

Foxc2 (ElDorado EntrezGene), Foxd1 (ElDorado EntrezGene),

Foxd2 (ElDorado EntrezGene), Foxd3 (ElDorado EntrezGene),

Foxd4 (ElDorado EntrezGene), Foxe1 (ElDorado EntrezGene),

Foxe3 (ElDorado EntrezGene), Foxf1 (ElDorado EntrezGene),

Foxf2 (ElDorado EntrezGene), Foxg1 (ElDorado EntrezGene),

Foxi1 (ElDorado EntrezGene), Foxj1 (ElDorado EntrezGene),

Foxj2 (ElDorado EntrezGene), Foxj3 (ElDorado EntrezGene),

Foxk1 (ElDorado EntrezGene), Foxk2 (ElDorado EntrezGene),

Foxl1 (ElDorado EntrezGene), Foxl2 (ElDorado EntrezGene),

Foxm1 (ElDorado EntrezGene), Foxn2 (ElDorado EntrezGene),

Foxn3 (ElDorado EntrezGene), Foxo1 (ElDorado EntrezGene),

Foxo3 (ElDorado EntrezGene), Foxo4 (ElDorado EntrezGene),

Foxo6 (ElDorado EntrezGene), Foxp1 (ElDorado EntrezGene),

Foxp2 (ElDorado EntrezGene), Foxp3 (ElDorado EntrezGene),

Foxp4 (ElDorado EntrezGene), Foxq1 (ElDorado EntrezGene),

Foxs1 (ElDorado EntrezGene), Hfh5 (ElDorado EntrezGene),

Hfh6 (ElDorado EntrezGene), Hfh7 (ElDorado EntrezGene)

HOMF mouse TFs:

Barhl1 (ElDorado EntrezGene), Barhl2 (ElDorado EntrezGene),

Barx1 (ElDorado EntrezGene), Barx2 (ElDorado EntrezGene),

Bsx (ElDorado EntrezGene), Hhex (ElDorado EntrezGene),

Hmx1 (ElDorado EntrezGene), Hmx2 (ElDorado EntrezGene),

Hmx3 (ElDorado EntrezGene), Lbx2 (ElDorado EntrezGene),

Msx1 (ElDorado EntrezGene), Msx2 (ElDorado EntrezGene),

Msx3 (ElDorado EntrezGene), Nobox (ElDorado EntrezGene),

Tlx1 (ElDorado EntrezGene), Tlx2 (ElDorado EntrezGene)

LHXF mouse TFs:

Isl1 (ElDorado EntrezGene), Isl2 (ElDorado EntrezGene),

Lhx1 (ElDorado EntrezGene), Lhx2 (ElDorado EntrezGene),

Lhx3 (ElDorado EntrezGene), Lhx4 (ElDorado EntrezGene),

Lhx5 (ElDorado EntrezGene), Lhx6 (ElDorado EntrezGene),

Lhx8 (ElDorado EntrezGene), Lhx9 (ElDorado EntrezGene),

Lmx1a (ElDorado EntrezGene), Lmx1b (ElDorado EntrezGene)

CEBP mouse TFS:

Cebpa (ElDorado EntrezGene), Cebpb (ElDorado EntrezGene),

Cebpd (ElDorado EntrezGene), Cebpe (ElDorado EntrezGene),

Cebpg (ElDorado EntrezGene), Cebpz (ElDorado EntrezGene)

EGRF mouse TFs:

Egr1 (ElDorado EntrezGene), Egr2 (ElDorado EntrezGene),

Egr3 (ElDorado EntrezGene), Egr4 (ElDorado EntrezGene),

Wt1 (ElDorado EntrezGene)

MYT1 mouse TFs:

Myt1 (ElDorado EntrezGene), Myt1l (ElDorado EntrezGene),

St18 (ElDorado EntrezGene)

HOXF mouse TFs:

Hoxa1 (ElDorado EntrezGene), Hoxa2 (ElDorado EntrezGene),

Hoxa3 (ElDorado EntrezGene), Hoxa4 (ElDorado EntrezGene),

Hoxa5 (ElDorado EntrezGene), Hoxa6 (ElDorado EntrezGene),

Hoxa7 (ElDorado EntrezGene), Hoxb1 (ElDorado EntrezGene),

Hoxb2 (ElDorado EntrezGene), Hoxb3 (ElDorado EntrezGene),

Hoxb4 (ElDorado EntrezGene), Hoxb5 (ElDorado EntrezGene),

Hoxb6 (ElDorado EntrezGene), Hoxb7 (ElDorado EntrezGene),

Hoxb8 (ElDorado EntrezGene), Hoxc4 (ElDorado EntrezGene),

Hoxc5 (ElDorado EntrezGene), Hoxc6 (ElDorado EntrezGene),

Hoxc8 (ElDorado EntrezGene), Hoxd1 (ElDorado EntrezGene),

Hoxd3 (ElDorado EntrezGene), Hoxd4 (ElDorado EntrezGene),

Hoxd8 (ElDorado EntrezGene), Nanog (ElDorado EntrezGene)

DMRT mouse TFs:

Dmrt1 (ElDorado EntrezGene), Dmrt2 (ElDorado EntrezGene),

Dmrt3 (ElDorado EntrezGene), Dmrta1 (ElDorado EntrezGene),

Dmrta2 (ElDorado EntrezGene), Dmrtb1 (ElDorado EntrezGene),

Dmrtc1a (ElDorado EntrezGene), Dmrtc1b (ElDorado EntrezGene),

Dmrtc2 (ElDorado EntrezGene)

CART mouse TFs:

Alx1 (ElDorado EntrezGene), Alx3 (ElDorado EntrezGene),

Alx4 (ElDorado EntrezGene), Arx (ElDorado EntrezGene),

Esx1 (ElDorado EntrezGene), Hesx1 (ElDorado EntrezGene),

Isx (ElDorado EntrezGene), Mixl1 (ElDorado EntrezGene),

Otp (ElDorado EntrezGene), Phox2a (ElDorado EntrezGene),

Phox2b (ElDorado EntrezGene), Prop1 (ElDorado EntrezGene),

Prrx1 (ElDorado EntrezGene), Prrx2 (ElDorado EntrezGene),

Rax (ElDorado EntrezGene), Rhox6 (ElDorado EntrezGene),

Shox2 (ElDorado EntrezGene), Uncx (ElDorado EntrezGene),

Vsx1 (ElDorado EntrezGene), Vsx2 (ElDorado EntrezGene)

MYBL mouse TFs:

Myb (ElDorado EntrezGene), Mybl1 (ElDorado EntrezGene),

Mybl2 (ElDorado EntrezGene)

PAX& mouse TFs:

Pax4 (ElDorado EntrezGene), Pax6 (ElDorado EntrezGene)

GCMF mouse TFs:

Gcm1 (ElDorado EntrezGene), Gcm2 (ElDorado EntrezGene)

ZF5F mouse TFs:

Zbtb14 (ElDorado EntrezGene)

**Supplementary References**

NOTE: These additional references provide published examples for functional transcriptional MORE-cassettes. MORE-cassettes were previously described in the literature as “frameworks”. However, since this expression elicited incorrect associations, we chose to rename “frameworks” to “Multiple Organized Regulatory Elements” (MOREs)-cassettes as this describes the basic structure. All > 900 entries of the Genomatix Module-library are MORE-cassettes (part of MatBase, all experimentally verified as synergistic or antagonistic), each with a reference of its own ([www.genomatix.de](http://www.genomatix.de/)). Additional individual publications (21), most with experimental verification of the defined MORE-cassettes:

1. Werner, T., Fessele, S., Maier, H., Nelson, P.J. (2003). Computer modeling of promoter organization as a tool to study transcriptional coregulation. FASEB J. 17, 1228-1237.

2. Fessele, S., Maier, H., Zischek, C., Nelson, P., J., Werner, T. (2002). Regulatory context is a crucial part of gene function. Trends Genet. 18, 60-63.

3. Masuda, K., Werner, T., Maheshwari, S., Frisch, M., Oh, S., Petrovics, G., May, K., Srikantan, V., Srivastava, S., Dobi, A. (2005). Androgen Receptor Binding Sites Identified by a GREF_GATA Model. J. Mol. Biol. 353, 763-771.

4. Klingenhoff, A., Frech, K., Quandt, K., Werner, T. (1999). Functional promoter modules can be detected by formal models independent of overall nucleotide sequence similarity, Bioinformatics 15, 180-186.

5. Werner T, Dombrowski SM, Zgheib C, Zouein FA, Keen HL, Kurdi M, Booz GW. (2013). Elucidating functional context within microarray data by integrated transcription factor-focused gene-interaction and regulatory network analysis. Eur Cytokine Netw. 24, 75-90.

6. Martini S, Nair V, Patel SR, Eichinger F, Nelson RG, Weil EJ, Pezzolesi MG, Krolewski AS, Randolph A, Keller BJ, Werner T, Kretzler M. (2013). From SNP to Transcriptional Mechanism: A Model for FRMD3 in Diabetic Nephropathy. Diabetes. 62, 2605 - 2612.

7. Coon, S.,L., Munson, P.,J., Cherukuri, P.,F., Sugden, D., Rath, M.,F., Møller, M., Clokie, S.,J., Fu, C., Olanich, M.,E., Rangel, Z., Werner, T.; NISC Comparative Sequencing Program, Mullikin, J.,C., Klein, D.,C. (2012) Circadian changes in long noncoding RNAs in the pineal gland. Proc Natl Acad Sci U S A. 109, 13319 - 13324.

8. Döhr, S., Klingenhoff, A., Maier, H., Hrabe de Angelis. M., Werner, T., Schneider, R. (2005). Linking disease-associated genes to regulatory networks via promoter organization. Nucleic Acids Res. 33, 864-872.

9. Seifert, M., Scherf, M., Epple, A., Werner, T. (2005). Multievidence microarray mining. Trends Genet. 21, 553-558.

10. Naschberger E, Werner T, Vicente AB, Guenzi E, Topolt K, Leubert R, Lubeseder-Martellato C, Nelson PJ, Sturzl M. (2004). A NF-kappaB motif and ISRE cooperate in the activation of guanylate binding protein-1 expression by inflammatory cytokines in endothelial cells. Biochem J. 20, 268 - 270.

11. Ziegler-Heitbrock, L Lötzerich, M., Schaefer, A, Werner, T.,. Frankenberger, M., Benkhart, E. (2003). IFNalpha induces the human IL-10 gene by recruiting both IRF-1 and Stat3, J Immunol. 171, 285-290.

12. Böhlk, S., Fessele, S., Mojaat, A., Miyamoto, N., G., Werner, T., Nelson, E., L., Schlöndorff, D., Nelson, P. (2000). ATF and Jun transcription factors, acting through an Ets / CRE promoter module, mediate lipopolysaccharide inducibility of the chemokine RANTES in monocytic Mono Mac 6 cells. Eur. J. Immunol. 30,1102-1112.

13. Cohen, C., D., Klingenhoff, A., Boucherot, A., Nitsche, A., Henger, A., Brunner, B., Schmid, H., Merkle, M., Saleem, M., A., Koller, K.-P., Werner, T., Gröne, H.-J., Nelson, P., J., Kretzler, M. (2006). Comparative promoter analysis allows de novo identification of specialized cell junction associated proteins. Proc. Natl. Acad. Sci. USA. 103, 5682 – 5687.
