# Supplementary figures and images for: Identification of genetic elements in metabolism by high-throughput mouse phenotyping
Source: Nat Commun. 2018 Jan 18;9:288. doi: 10.1038/s41467-017-01995-2 (PMC5773596; doi:10.1038/s41467-017-01995-2)

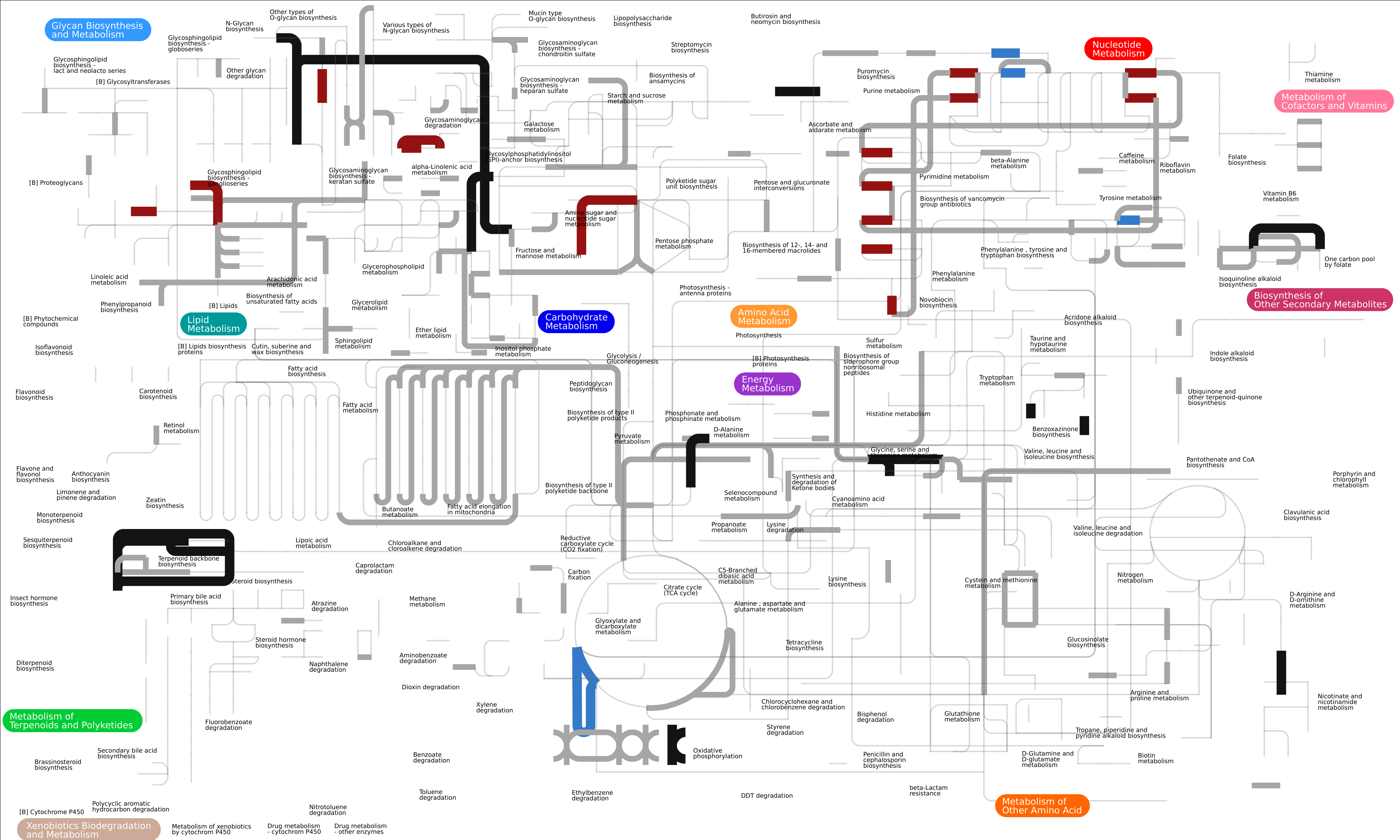

Supplement: Supplementary file 11 — Supplementary Data 9 [file 41467_2017_1995_MOESM11_ESM.pdf]

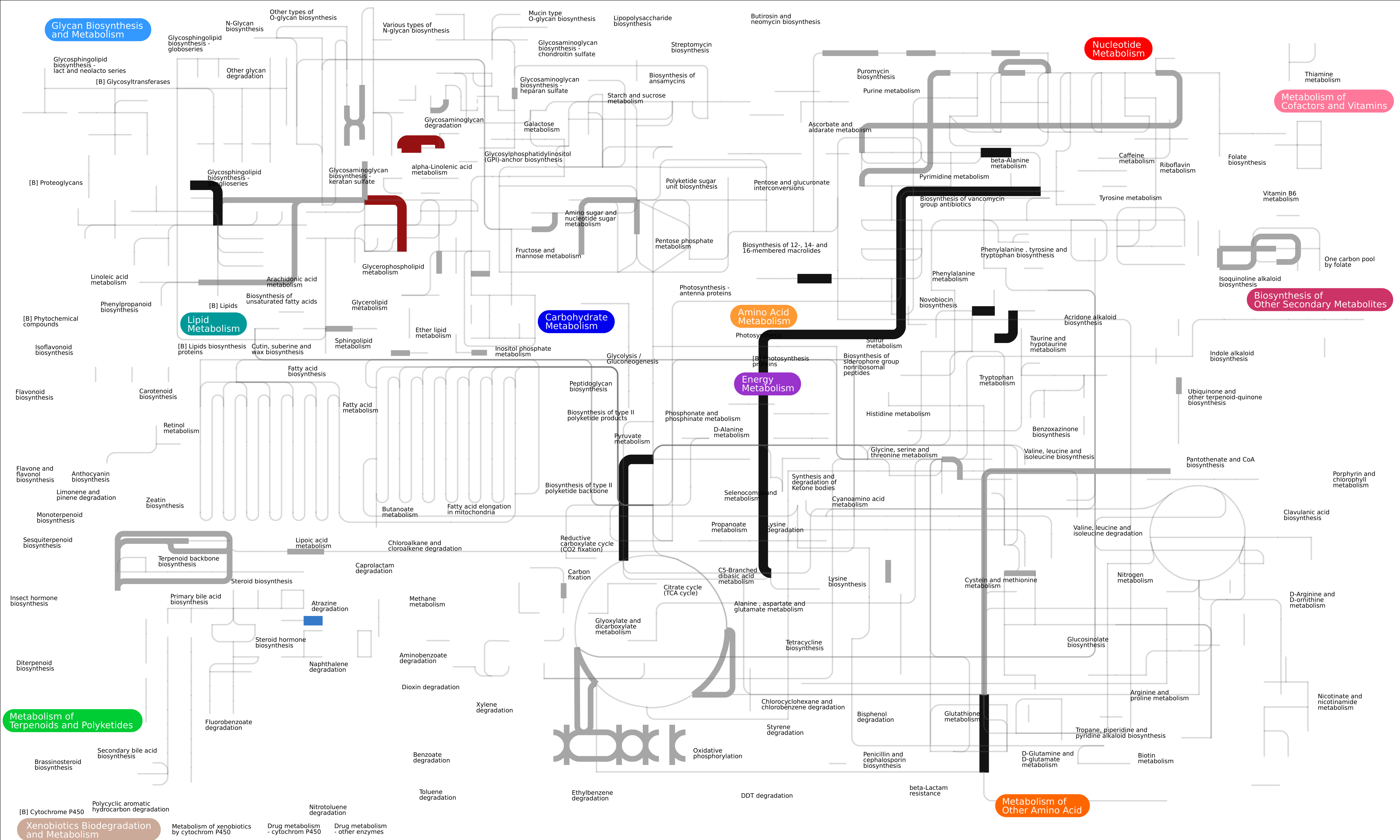

Supplement: Supplementary file 16 — Supplementary Data 14 [file 41467_2017_1995_MOESM16_ESM.pdf]
