# Supplementary material for: Identification of genetic elements in metabolism by high-throughput mouse phenotyping
Source: Nat Commun. 2018 Jan 18;9:288. doi: 10.1038/s41467-017-01995-2 (PMC5773596; doi:10.1038/s41467-017-01995-2)
Supplement: Supplementary file 12 — Supplementary Data 10 [file 41467_2017_1995_MOESM12_ESM.pdf]

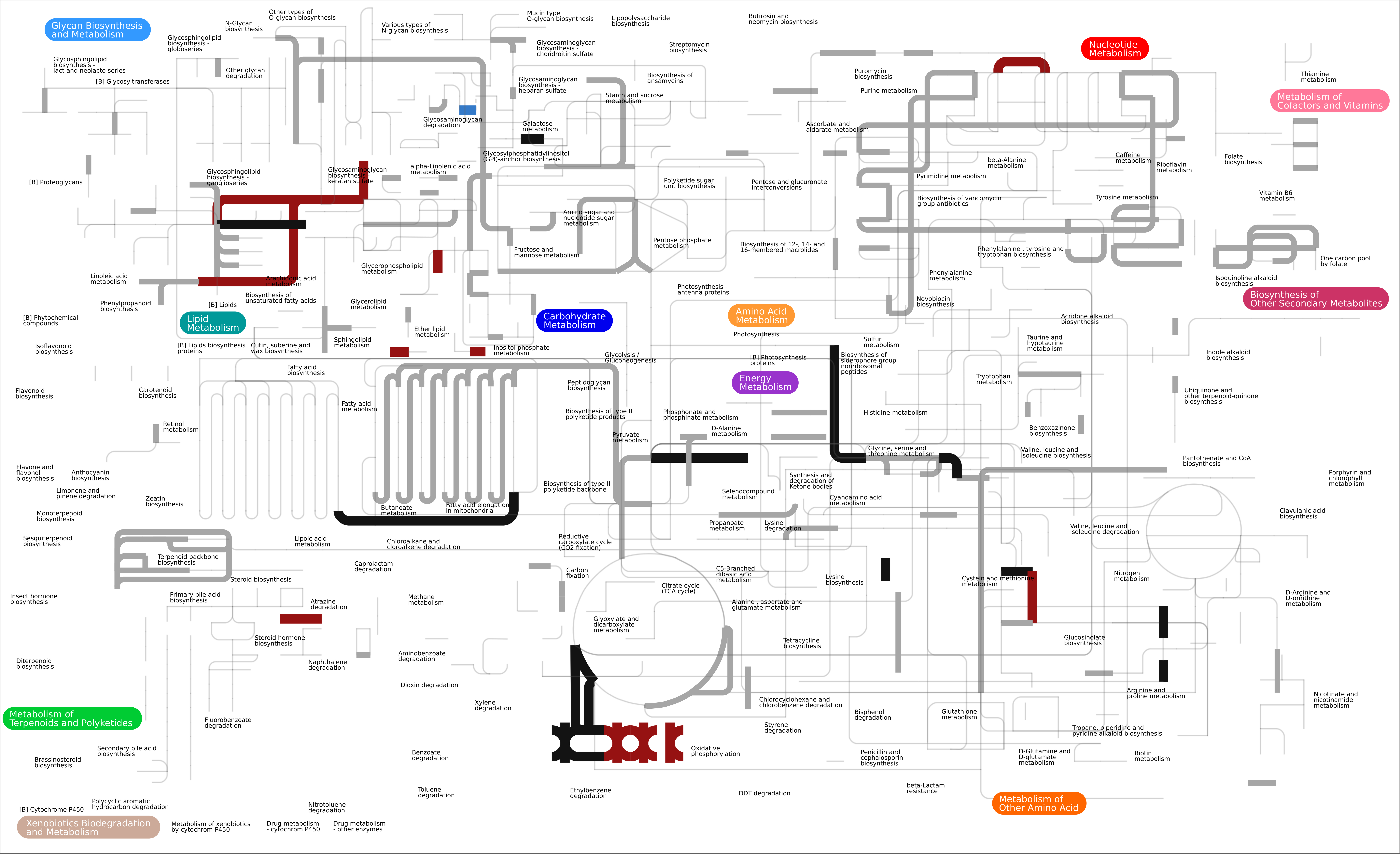

Glycan Biosynthesis and Metabolism
Glycosphingolipid biosynthesis - lact and neolacto series
[B] Glycosyltransferases
Glycosphingolipid biosynthesis - globoseries
N-Glycan biosynthesis
Other types of O-glycan biosynthesis
Other glycan degradation
Various types of N-glycan biosynthesis
Mucin type O-glycan biosynthesis
Lipopolysaccharide biosynthesis
Butirosin and neomycin biosynthesis
Glycosaminoglycan biosynthesis - chondroitin sulfate
Streptomycin biosynthesis
Nucleotide Metabolism
Thiamine metabolism
Puromycin biosynthesis
Glycosaminoglycan biosynthesis - heparan sulfate
Biosynthesis of ansamycins
Purine metabolism
Metabolism of Cofactors and Vitamins
Starch and sucrose metabolism
Glycosaminoglycan degradation
Galactose metabolism
Ascorbate and aldarate metabolism
Glycosylphosphatidylinositol (GPI)-anchor biosynthesis
Caffeine metabolism
Riboflavin metabolism
Folate biosynthesis
beta-Alanine metabolism
[B] Proteoglycans
Glycosphingolipid biosynthesis - ganglioseries
Glycosaminoglycan biosynthesis - keratan sulfate
alpha-Linolenic acid metabolism
Polyketide sugar unit biosynthesis
Pentose and glucuronate interconversions
Pyrimidine metabolism
Biosynthesis of vancomycin group antibiotics
Tyrosine metabolism
Vitamin B6 metabolism
Amino sugar and nucleotide sugar metabolism
Pentose phosphate metabolism
Biosynthesis of 12-, 14- and 16-membered macrolides
Fructose and mannose metabolism
Phenylalanine , tyrosine and tryptophan biosynthesis
One carbon pool by folate
Glycerophospholipid metabolism
Linoleic acid metabolism
Arachidonic acid metabolism
Phenylalanine metabolism
Isoquinoline alkaloid biosynthesis
Photosynthesis - antenna proteins
Biosynthesis of unsaturated fatty acids
[B] Lipids
Glycerolipid metabolism
Novobiocin biosynthesis
Biosynthesis of Other Secondary Metabolites
Phenylpropanoid biosynthesis
[B] Phytochemical compounds
Lipid Metabolism
Carbohydrate Metabolism
Amino Acid Metabolism
Acridone alkaloid biosynthesis
Ether lipid metabolism
Photosynthesis
Sphingolipid metabolism
Sulfur metabolism
Taurine and hypotaurine metabolism
Isoflavonoid biosynthesis
[B] Lipids biosynthesis proteins
Cutin, suberine and wax biosynthesis
Inositol phosphate metabolism
Glycolysis / Gluconeogenesis
[B] Photosynthesis proteins
Biosynthesis of siderophore group nonribosomal peptides
Indole alkaloid biosynthesis
Fatty acid biosynthesis
Energy Metabolism
Tryptophan metabolism
Flavonoid biosynthesis
Carotenoid biosynthesis
Peptidoglycan biosynthesis
Ubiquinone and other terpenoid-quinone biosynthesis
Fatty acid metabolism
Biosynthesis of type II polyketide products
Phosphonate and phosphinate metabolism
Histidine metabolism
Retinol metabolism
Pyruvate metabolism
D-Alanine metabolism
Benzoxazinone biosynthesis
Glycine, serine and threonine metabolism
Valine, leucine and isoleucine biosynthesis
Pantothenate and CoA biosynthesis
Flavone and flavonol biosynthesis
Anthocyanin biosynthesis
Synthesis and degradation of Ketone bodies
Porphyrin and chlorophyll metabolism
Biosynthesis of type II polyketide backbone
Limonene and pinene degradation
Selenocompound metabolism
Zeatin biosynthesis
Cyanoamino acid metabolism
Butanoate metabolism
Fatty acid elongation in mitochondria
Monoterpenoid biosynthesis
Clavulanic acid biosynthesis
Propanoate metabolism
Lysine degradation
Valine, leucine and isoleucine degradation
Sesquiterpenoid biosynthesis
Lipoic acid metabolism
Chloroalkane and cloroalkene degradation
Reductive carboxylate cycle (CO2 fixation)
Terpenoid backbone biosynthesis
Caprolactam degradation
Carbon fixation
C5-Branched dibasic acid metabolism
Nitrogen metabolism
Steroid biosynthesis
Lysine biosynthesis
Cystein and methionine metabolism
Citrate cycle (TCA cycle)
Insect hormone biosynthesis
Primary bile acid biosynthesis
Atrazine degradation
Methane metabolism
D-Arginine and D-ornithine metabolism
Alanine , aspartate and glutamate metabolism
Glyoxylate and dicarboxylate metabolism
Steroid hormone biosynthesis
Glucosinolate biosynthesis
Tetracycline biosynthesis
Aminobenzoate degradation
Naphthalene degradation
Diterpenoid biosynthesis
Dioxin degradation
Arginine and proline metabolism
Nicotinate and nicotinamide metabolism
Xylene degradation
Chlorocyclohexane and chlorobenzene degradation
Glutathione metabolism
Bisphenol degradation
Metabolism of Terpenoids and Polyketides
Fluorobenzoate degradation
Styrene degradation
Tropane, piperidine and pyridine alkaloid biosynthesis
Oxidative phosphorylation
Secondary bile acid biosynthesis
Benzoate degradation
Penicillin and cephalosporin biosynthesis
D-Glutamine and D-glutamate metabolism
Biotin metabolism
Brassinosteroid biosynthesis
Toluene degradation
Ethylbenzene degradation
DDT degradation
beta-Lactam resistance
Polycyclic aromatic hydrocarbon degradation
[B] Cytochrome P450
Nitrotoluene degradation
Metabolism of Other Amino Acid
Xenobiotics Biodegradation and Metabolism
Metabolism of xenobiotics by cytochrom P450
Drug metabolism - cytochrom P450
Drug metabolism - other enzymes
